# Attachment Site Selection and Identity in Bxb1 Serine Integrase-Mediated Site-Specific Recombination

**DOI:** 10.1371/journal.pgen.1003490

**Published:** 2013-05-02

**Authors:** Shweta Singh, Pallavi Ghosh, Graham F. Hatfull

**Affiliations:** Department of Biological Sciences, University of Pittsburgh, Pittsburgh, Pennsylvania, United States of America; Duke University Medical Center, United States of America

## Abstract

Phage-encoded serine integrases mediate directionally regulated site-specific recombination between short *attP* and *attB* DNA sites without host factor requirements. These features make them attractive for genome engineering and synthetic genetics, although the basis for DNA site selection is poorly understood. Here we show that *attP* selection is determined through multiple proofreading steps that reject non-*attP* substrates, and that discrimination of *attP* and *attB* involves two critical site features: the outermost 5–6 base pairs of *attP* that are required for Int binding and recombination but antagonize *attB* function, and the “discriminators” at positions −15/+15 that determine *attB* identity but also antagonize *attP* function. Thus, although the attachment sites differ in length and sequence, only two base changes are needed to convert *attP* to *attL*, and just two more from *attL* to *attB*. The opposing effect of site identifiers ensures that site schizophrenia with dual identities does not occur.

## Author Summary

Site-specific recombinases catalyze recombination between two specific DNA sites to generate the products of recombination. The Integrase encoded by mycobacteriophage Bxb1 is a member of the serine-recombinase family and catalyzes strand exchange between *attP* and *attB*, the attachment sites for the phage and bacterial host, respectively. Although the DNA sites are relatively small (<50 bp), the reaction is highly selective for these sites and is also strongly directional. Here, we address the question of what sequences within *attP* are required for it to act as an *attP* site and identify the key sequence features that are required not just for Integrase binding but also for synapsis and post-synapsis events. We also have identified the key determinants of *attP* and *attB* identity, and although the sites are different in sequence and length, they can be interconverted with just two base changes in each of the half sites.

## Introduction

**Figure 1:**
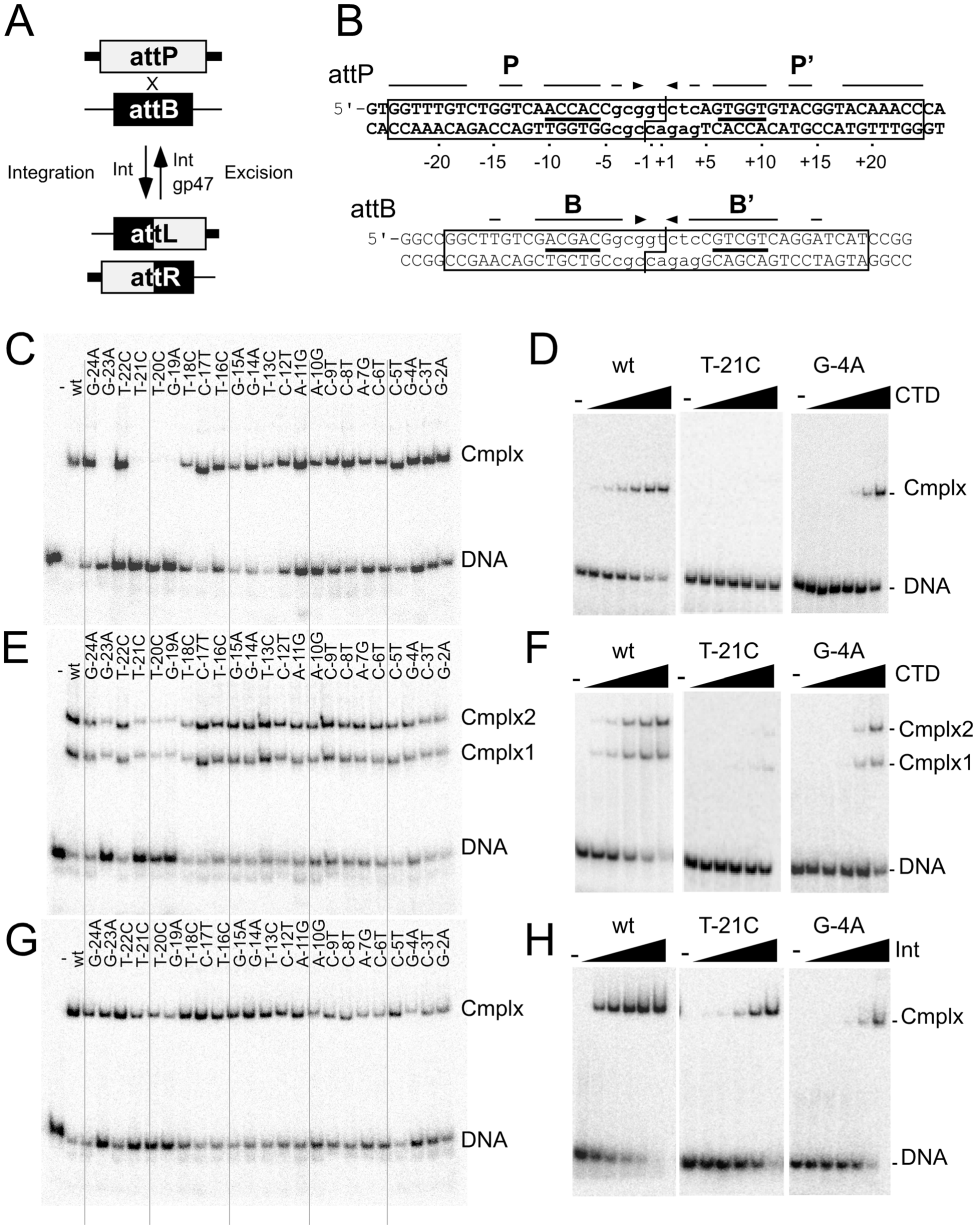
Effect of P half site mutations on Bxb1 Int and CTD binding. (A) Integration and excision mediated by Bxb1 serine-integrase. Attachment sites *attP* and *attB* are substrates for Integrase (Int) mediated site-specific recombination to generate attachment sites *attL* and *attR* as products. *attL* and *attR* are used as substrates for integrase-mediated excision but only in the present of the recombination directionality factor (RDF), Bxb1 gp47. The products of excisive recombination are *attP* and *attB*. (B) Sequences of Bxb1 *attP* and *attB*. The minimally required sequences are boxed, and the cleavage sites around the central dinucleotide are shown (|―|). Interrupted arrows above each sequence show symmetrically conserved base pairs, and lines show the position of the conserved 5′-ACNAC motif. Common core sequences are shown in lower case. The numbering scheme for *attP* base positions is shown. (C). Separation of complexes formed between CTD (1215 nM) and a 50 bp half-site DNA containing the P half site. (D) Titration of CTD binding to the T-21C and G-4A half site substrates; additional substrates are shown in Figure S1A. Concentrations of CTD are 0, 5, 15, 45, 135, 405 and 1215 nM. (E) Separation of complexes formed between CTD (1215 nM) and a 50 bp full-site *attP* DNA containing the P half site mutations. (F) Titration of CTD binding to the T-21C and G-4A full-site substrates; additional substrates are shown in Figure S1B. Concentrations of CTD are 0, 15, 45, 135, 405 and 1215 nM. (G) Separation of complexes formed between Int (405 nM) and *attP* mutant substrates as indicated. (H) Titration of Int binding to the T-21C and G-4A full-site substrates; additional substrates are shown in Figure S1C. Int concentrations used are 0, 15, 45, 135, 405 and 1215 nM.

Establishment of lysogeny by temperate bacteriophages typically involves site-specific integration of the phage genome into the host chromosome. Integration is catalyzed by a phage-encoded Integrase protein (Int) mediating site-specific recombination between phage and bacterial attachment sites (*attP* and *attB* respectively), and generates attachment site junctions (*attL* and *attR*) as products of the reaction (Figure 1A) [1]. There are two major classes of phage integrases – corresponding to the tyrosine- and serine-recombinase families – that use distinct mechanisms of strand exchange and have different site and protein requirements [2]. The tyrosine integrases typically utilize a relatively large *attP* site (∼250 bp) containing multiple binding sites for integrase, a host-encoded integration host factor, and a recombination directionality factor (RDF) that binds and bends DNA to confer directionality of recombination [3], [4]. In contrast, serine-integrases use simple attachment sites (<50 bp), have no host factor requirements, and the RDF does not act through direct binding to DNA [5]–[7]. Because of these features, serine-integrases function well in heterologous systems, making them attractive for genome engineering in human, mouse, drosophila, and malarial cells [8]–[11], as well as powerful switches for synthetic genetic circuits and microbial data storage systems [12], [13].

The serine-integrase (Int) coded by mycobacteriophage Bxb1 is relatively large (500 aa) and contains an N-terminal catalytic domain (∼150 aa) common to all serine-recombinases, and a C-terminal domain (CTD; 350 aa) that binds DNA [14], [15]. The *attP* and *attB* sites are small and have different length requirements, 48 bp for *attP* and 38 bp for *attB*. Strand cleavage occurs about an asymmetric central dinucleotide within a protein-mediated synaptic complex, followed by rotation and religation [16]. Recombination is highly selective for the cognate attachment sites, *attP* and *attB* for integration, and *attL* and *attR* for excision, and is strongly directional, such that excision only occurs in the presence of the recombination directionality factor (RDF), Bxb1 gp47 (Figure 1A) [7]. *attP* and *attB* are functionally symmetrical such that the central dinucleotide is the sole determinant of integration polarity [15], and the sequences of both sites are partially symmetric, although outside of an 8 bp common core there is only limited sequence similarity (Figure 1B). Bxb1 Int binds as a dimer to *attP* and *attB* with similar affinities (Kd 70 nM), but somewhat tighter to *attL* and *attR* (Kd 15 nM); CTD binds as a monomer to each half site with similar affinities for the B, B′ and P half sites (∼120 nM), and a somewhat lower affinity for the P′ half site [17]. These general features are shared by other well-studied serine integrase systems [5], [18]–[24].

Selection of cognate sites that support recombination involves multiple steps in the reaction [25], [26]. DNA binding is required but is not sufficient, and in the absence of the RDF, synapsis only occurs between Int dimers bound at *attP* and *attB* [15], [27]. Because synapsis is protein-mediated, Int presumably adopts different configurations when bound at different *att* sites with synapsis requiring compatible configurations [17], [27]. However, synapsis of *attL* and *attR* in the presence of the RDF is orientation dependent, suggesting that an Int protomer bound at a B-type half site (either B or B′) can only productively interact with a P-type half site (P or P′) [26]. Substitutions in the *attB* site of φC31 show that specific DNA sequences are also important for post-synaptic events [25].

Here, we investigate what specific sequences are required for Bxb1 Int to recognize its *attP* site and to functionally distinguish *attP* and *attB*. We show that there are two critical site components. One is the outermost flanks of *attP* that are required for Int binding and for recombination, but which also prevent usage as *attB*. The second is a key discriminator position at positions −15 and +15 where a T∶A/A∶T base pair (in B and B′ half sites respectively) is required for both Int binding and recombination as an *attB* site, but which interferes with *attP* functionality. The identities of *attP* and *attB* are mutually exclusive, but they can be interconverted with mutations in the critical discriminator and flanking motifs.

## Results

### Impact of *attP* mutations on Bxb1 Int and CTD binding

To determine the sequence contributions of Int binding to *attP*, we initially constructed a series of altered half-site substrates with transition mutations at each of 23 positions within the P half site, and examined the binding of the Bxb1 Integrase CTD (Figure 1C, Figure S1, Table 1). Substitutions at four positions (−19, −20, −21, and −23) are strongly deleterious to CTD binding and no complex is observed even at the highest protein concentrations tested (Figure 1D, Figure S1A). These positions are all at the extreme flank of the P half site and – with the exception of position −19 – are outside of the corresponding minimal site requirements for *attB* (Figure 1B). They also are all symmetrically conserved between the P and P′ half sites (Figure 1B). Substitutions at most of the other positions in the P site also impair CTD binding but to lesser extents (Figure 1C, 1D, Figure S1A, Table 1). A summary of all mutant site activities is shown in Table 2.

**Table 1:** Binding affinities of Bxb1 Int and Int-CTD for *attP* mutants.

Mutation^1^	Kd of CTD binding to P half site (nM)^2^	Kd of CTD binding to full site (nM)^3^	Kd of Int binding to full site (nM)^3^
Wild type	140	150	60
G-2A	500	340	160
C-3T	970	490	NT
G-4A	1050	710	460
C-5T	600	200	NT
C-6T	990	760	350
A-7G	990	520	480
C-8T	320	280	NT
C-9T	840	290	360
A-10G	>1215	820	540
A-11G	700	670	NT
C-12T	120	60	NT
T-13C	300	240	NT
G-14A	260	180	NT
G-15A	320	310	NT
T-16C	560	490	NT
C-17T	70	100	NT
T-18C	900	450	145
G-19A	No binding	>1215	550
T-20C	No binding	>1215	575
T-21C	No binding	>1215	380
T-22C	430	120	NT
G-23A	No binding	>1215	285
G-24A	120	135	320
G-2A, C+2T	-	340	65
G-4A, C+4T	-	1040	625
C-6T, G+6A	-	>1215	460
C-9T, G+9A	-	920	240
A-10G, T+10C	-	>1215	520
T-18C, A+18G	-	>1215	105
G-19A, C+19T	-	No binding	>1215
T-20C, A+20G	-	No binding	>1215
T-21C, A+21G	-	No binding	>1215
G-23A, C+23T	-	No binding	1040
G-24A, C+24T	-	740	65

1 Mutations are denoted as the wild-type base, the position, and the altered base.

2 50 bp P half-site substrates contain positions −24 to +4.

3 50 bp full site substrates.

No Binding: No binding was observed even at the highest concentration tested (1215 nM).

NT: Not tested.

**Table 2:** Summary of mutant *attP* site behaviors.

Mutation	CTD binding to half site DNA^1^	CTD binding to full site DNA^2^	Int binding to full site DNA^3^	Recombn^4^	Synapsis^5^	Cleavage^6^
Wild type	+++	+++	+++	+++	+++	+++
G-2A	++	++	++	+++	+++	+
C-3T	+	++	++	+++	NT	NT
G-4A	+	+	+	+	+++	++
C-5T	++	+++	+++	+++	NT	NT
C-6T	+	+	+	+++	+++	++
A-7G	+	++	++	+++	+++	++
C-8T	++	+++	+++	+++	NT	NT
C-9T	+	+++	++	+++	+++	++
A-10G	+	+	+	+++	+++	++
A-11G	+	+	+	+++	NT	NT
C-12T	+++	+++	+++	+++	NT	NT
T-13C	+++	+++	+++	+++	NT	NT
G-14A	+++	+++	+++	+++	NT	NT
G-15A	++	++	++	+++	NT	NT
T-16C	++	++	++	+++	NT	NT
C-17T	+++	+++	+++	+++	NT	NT
T-18C	+	++	+++	+++	+++	++
G-19A	−	+	+	+	+++	++
T-20C	−	+	+	+	++	++
T-21C	−	+	+	+	++	+++
T-22C	++	+++	+++	++	NT	NT
G-23A	−	+	++	+	+++	+++
G-24A	+++	+++	+++	+++	NT	NT
C+2T	+++	+++	+++	+++	+++	++
C+4T	+	+++	++	+	+	+
G+6A	++	++	++	+++	+++	+++
G+9A	++	++	+	+++	+++	+++
T+10C	++	++	++	+++	+++	+++
A+18G	+	++	++	+++	+++	++
C+19T	+	++	++	+	+++	+
A+20G	+	++	++	+	++	+
A+21G	−	++	++	+	++	+
C+23T	+	++	++	+	+++	+++
G-2A, C+2T	N/A	++	+++	+++	+++	+++
G-4A, C+4T	N/A	+	+	−	−	−
C-6T, G+6A	N/A	+	+	+++	+++	+++
C-9T, G+9A	N/A	+	++	+++	+++	+++
A-10G, T+10C	N/A	+	+	+++	NT	+++
T-18C, A+18G	N/A	+	+++	+++	+++	+++
G-19A, C+19T	N/A	−	−	+	−	−
T-20C, A+20G	N/A	−	−	+	−	−
T-21C, A+21G	N/A	−	−	−	−	−
G-23A, C+23T	N/A	−	−	+	+	+
G-24A, C+24T	N/A	+	+++	+++	NT	NT

1 Scores reflect Kd's: no binding, −; 140–300 nM; +++, 301–600, ++; >600 nM, +.

2 Scores reflect apparent Kd's: no binding, −; 140–300 nM; +++, 301–600, ++; >600 nM, +.

3 Values reflect Kd's: no binding, −; 60–150 nM; +++, 151–300, ++; >300 nM, +.

4 Values reflect recombination relative to wild-type (+++): no recombination, −; good; +++, fair, ++; poor, +.

5 Values reflect synapsis relative to wild-type (+++): no synapsis, −; good; +++, fair, ++; poor, +.

6 Values reflect cleavage relative to wild-type (+++): no cleavage, −; good; +++, fair, ++; >poor, +.

N/A: Not Applicable; NT: Not Tested.

See text, figures, and Figures S1, S2, S3, S4 for further details.

We extended this analysis to determine how Int CTD binds to full site *attP* substrates containing P site substitutions (Figure 1E, 1F). CTD binding to wild-type *attP* has a somewhat unusual pattern in that two complexes are formed (complex 1 and complex 2; Figure 1E) whose identities are not clear, and it is presumed that the slower migrating complex (complex 2) contains CTD protomers bound to both half sites, and that complex 1 has only a single CTD protomer. However, higher concentrations of CTD do not drive *attP* DNA from complex 1 into complex 2 unless there is a nick at the center of the site [17], suggesting that DNA rigidity contributes to interference between CTD protomers binding to both half sites. In general, the impact of P-substitutions on CTD binding to these substrates reflects those seen with half-site DNAs (Figure 1E, 1F, Figure S1B) although the −19, −20, −21 and −23 substitutions have a more modest impact suggesting that binding of CTD to the P′ half site can stimulate CTD with mildly cooperative binding to the P half site.

Full length Int binds cooperatively as a dimer to *attP* forming a single complex (Figure 1G, 1H, Figure S1C), and substitutions in the P component generally have only mildly reduced binding, including the flank positions that strongly impair CTD binding. No single base substitution reduces Int binding by more than about 10-fold (Figure 1G, 1H, Figure S1C, Table 1).

**Figure 2:**
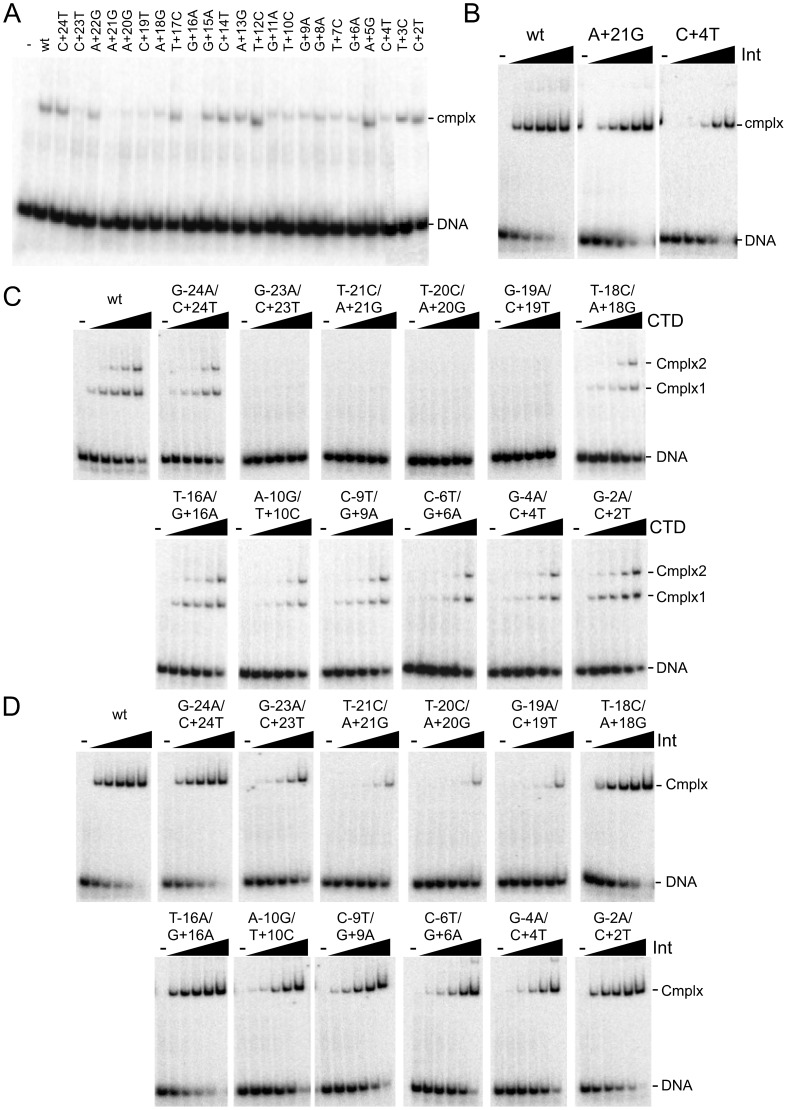
Binding of Int and CTD to P′ and double mutant substrates. (A) Separation of complexes formed between CTD (1215 nM) and P′ half site substrates containing single substitutions as shown. Examples of CTD binding titrartions to full site *attP* substrates containing P′ mutations are shown in Figure S2A. (B) Binding titrations of Int to A+21G and C+4T mutant *attP* DNA substrates containing P′ mutations as indicated. Int concentrations are 0, 15, 45, 135, 405 and 1215 nM. Other mutant substrates are shown in Figure S2B. (C and D) Binding titrations of CTD (C) and Int (D) to *attP* substrates containing mutations in both half sites as indicated. CTD and Int concentrations are 0, 15, 45, 135, 405 and 1215 nM.

A similar series of binding experiments were performed with substitutions in the P′ arm (Figure 2). The cognate mutations generally have similar effects on binding to a P′ half site as to the P half site, although the binding to the wild-type site is relatively weak and determining affinities is more difficult (Figure 2A). Substitutions at positions +23 and +21 are the most deleterious to binding, with lesser effects by other mutations. In the context of the full *attP* site, the substitutions primarily influence the formation of complex 2 by CTD (Figure S2A), and none of the mutants tested has a substantial impact on Int binding (Figure 2B, Figure S2B, Table S1).

Finally, we examined the impact of double substitutions at symmetrically related positions in both half sites (Figure 2C, 2D, Table 1). Double substitutions at positions −23/+23, −21/+21, −20/+20, −19/+19 strongly interfere with CTD binding (Figure 2C), and substantially reduced Int binding (Figure 2D). Some of the double mutants – such as −23/+23 have poor CTD binding – but Int itself binds reasonably well. Overall, these binding data illustrate the important roles of the extreme flanking sequences for recognition of *attP* by Int, and the important but lesser contributions at a large number of positions in the inner part of the site.

### Impact of *attP* site mutations on recombination

**Figure 3:**
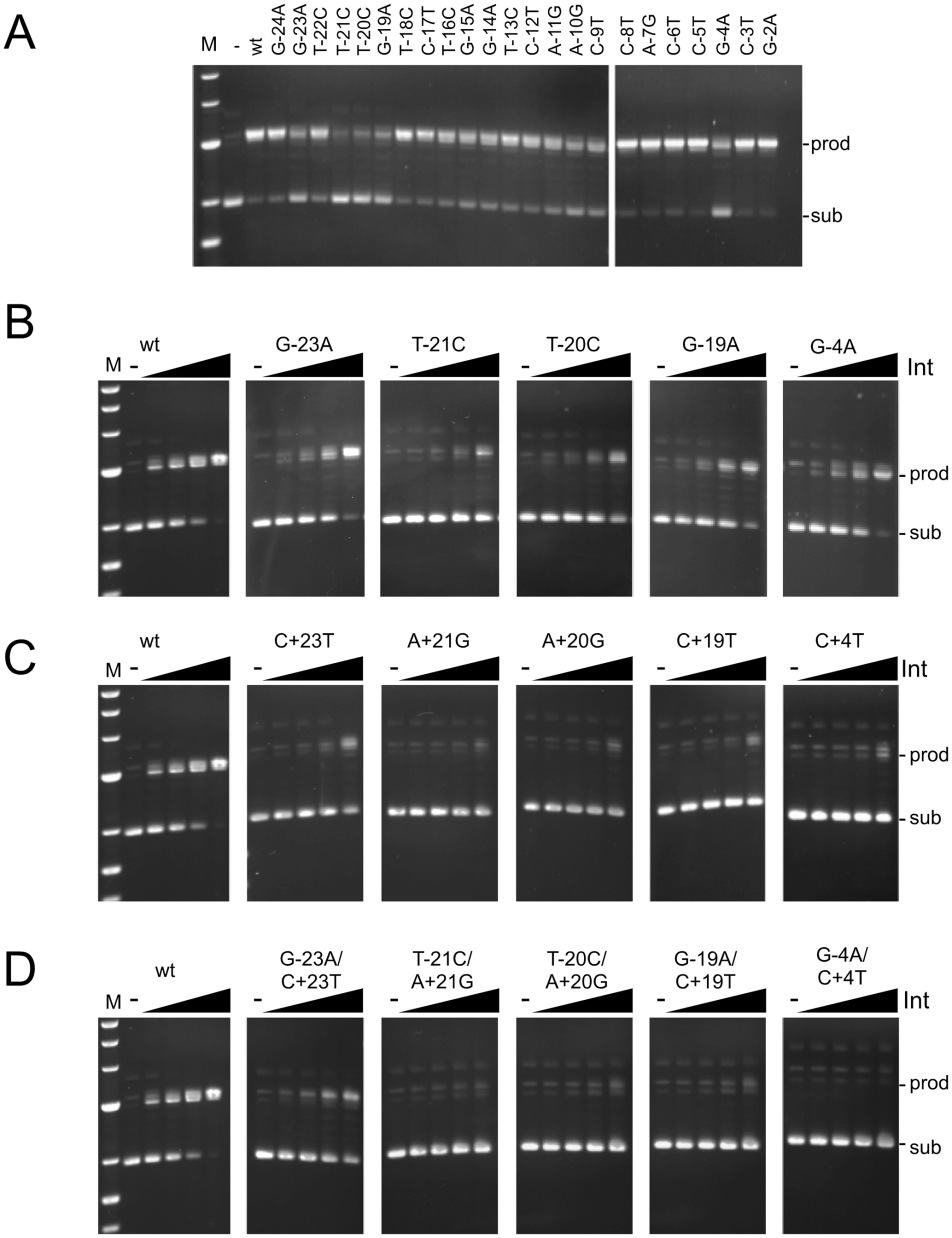
Recombination potential of mutant *attP* substrates. (A) Recombination activities are shown for substrates having single mutations (as indicated) in the P-site of *attP* in the presence of gpInt (150 nM). (B–D) Recombination of P-site mutants (B), P′ mutants (C) and double mutants (D) as indicated. Recombination assays were performed using *attB* plasmid (pMOS) and 50 bp *attP* substrates. The concentrations of Int used are 0, 37.5, 75, 150 and 300 nM. Positions of substrate (sub) and product (prod) DNAs are indicated. Additional substrates are shown in Figure S3.

We surveyed all of the P-mutants (in the *attP* context) for their ability to support integrative recombination (Figure 3A), and analyzed subsets of these as well as P′ mutants and double mutants in further detail (Figure 3B, 3C, 3D, Figure S3). In general, most of the single substitutions in the *attP* flanks (−23, −21, −20, −19, +19, +20, +21, +23) are deleterious for recombination, even though Int binding to most of these substrates is only mildly affected (Figure 3B, 3C). Similarly, single substitutions at −4 and +4 also impair recombination (Figure 3B, 3C), even though Int binds reasonably well (Figure 1H, Figure 2B, Table 1, Table S1). Double mutants that strongly interfere with Int binding (e.g. −21/+21, −20/+21, −19/+19) not surprisingly are strongly defective in recombination (Figure 3D, Figure S3C). The poor recombination of the −4/+4 double mutant reflects the behaviors of the single substitutions at position 4, and Int binds reasonably well to the double mutant (Figure 3D, Figure 2D). These observations show that the *attP* sequence influences not only Int binding, but is also important for subsequent steps in the reaction, either synapsis or post-synaptic events. Moreover, there are two distinct types of effect: the *attP* flank sequences that are required for CTD recognition but are also important for recombination (although we cannot rule out that the recombination defect is largely a consequence of poor Int binding), and the −4/+4 positions that have a modest contribution to CTD binding, but are critical for recombination. A summary of mutant site activities is shown in Table 2.

### Effect of *attP* mutations on synapsis and cleavage

**Figure 4:**
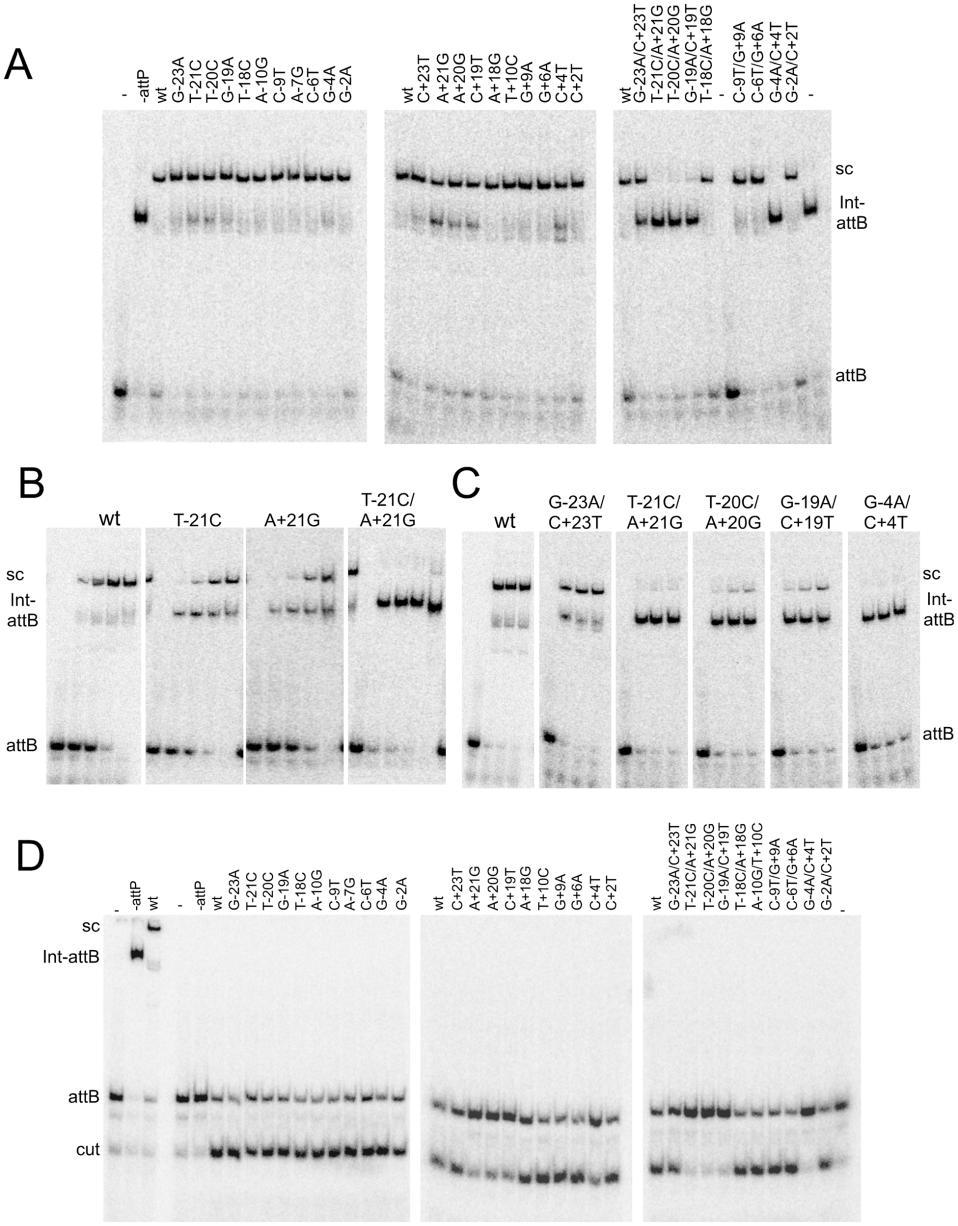
Synaptic complex formation by mutant *attP* substrates. (A) Mutant *attP* substrates with point mutations in the P-site (left most panel) or P′-site (middle panel) and double mutations (one in P and one in P′-site, right panel), show synaptic complex formation with the radiolabeled suicide *attB* substrate in presence of 300 nM of Int. (B) The *attP* mutants are incubated with radiolabeled suicide *attB* substrate and varying concentration of gpInt for synaptic complex formation. The concentrations of gpInt are 0, 45, 135, 405, 1215 nM. Additional substrates are shown in Figure S4. (C) Time-dependence of synaptic complex formation with mutant *attP* substrates. The Int concentration is 405 nM and incubation times are 1, 2 and 3 hours. (D) Cleavage reactions were done similarly to synaptic complex formation as in (A), but were treated with Proteinase K and SDS before running on an 8% (w/v) polyacrylamide gel. Position of cleaved product (cut) is indicated.

For those substrates to which Int binding is observed but recombination is impaired, the defect could be at the requirement for synaptic complex formation between *attP* and *attB*, or a post-synaptic event involving strand cleavage, rotation or rejoining. To examine this, we tested mutant substrates for their ability to form synaptic complexes with an *attB* suicide substrate (Figure 4) [7]. Substrates with single mutations in either P or P′ that support Int binding at reasonable levels (Figure 1, Figure 2, Table 1, Table S1) generally show good synaptic complex formation, with milder defects in *attP* flank mutants as well at the −4 and +4 positions (Figure 4A, 4B, Figure S4). In general, mutants with mild defects in synaptic complex formation (T-21C, T-20C, G-4A, A+21G, A+20G, C+19T, C+4T) are also strongly defective in recombination, even though Int binds reasonably well to most of these substrates (Figure 4A, 4B, 4C, Figure S4). But even the A+21G substrate – to which Int binds normally (Figure 2B) – forms good synaptic complexes at high Int concentrations (Figure 4B), even though recombination is strongly impaired (Figure 3C). Among the double mutants, the substitutions at positions 21, 20 and 19 fail to form synaptic complexes (Figure 4A, 4B, 4C) but this reflects the strong defects in Int binding. Extended incubation promotes synapsis for the −/+19 mutant (Figure 4C). In contrast, the strong recombination defect of the −4/+4 mutant appears to result from strong inhibition of synapsis.

Cleavage assays show that mutants with single substitutions at the +21, +20 and +19 positions are strongly defective in cleavage (Figure 4D), even though they can form synaptic complexes – albeit inefficiently. In contrast, other single substitutions – primarily in the P half of *attP* – appear to undergo cleavage reasonably well. The difference between the cleavage capacity of P and P′ mutants could reflect the asymmetry of the *attB* suicide substrate (in which only the top strand contains a gap) and only cleavage of the bottom strand is required to generate a dsDNA cleaved product (Figure 4D). These data are thus consistent with the interpretation that the Int protomer bound to the P′ half site is specifically responsible for cleavage of the bottom strand. Nonetheless, these observations show that single base substitutions (such as T-21C) can inhibit post-cleavage events in the reaction, such as rotation or rejoining (Figure 4D). A summary of all mutant site activities is shown in Table 2.

Taken together, these observations show that there are multiple stages in the integration reaction where the sequence of *attP* influences recombination. These can be thought of as a series of proofreading events in which the site sequence is interpreted for whether it is permissive for recombination. In the initial binding stage for example, the T-21C/A+21G mutant is strongly defective in binding and recombination does not occur. At the next step of synapsis, a mutant such as G-4A/C+4T is bound reasonably well by Int (Figure 2D), but this mutant is rejected for synapsis (Figure 4C). But even if a mutant such as T-21C is bound by Int, synapses with *attB* and undergoes cleavage, it is impaired for rotation or religation. This is consistent with a model in which site-selection involves the formation of specific conformations of protein-DNA complexes, and inappropriate conformations prevent not only synapsis but also post-synaptic events.

### Site identity: What makes *attP*, *attP*, and *attB*, *attB*?

The experiments described above identify the roles of specific base pairs in *attP* that enable it to recombine with *attB*. The sequences at the extreme flanks of *attP* play critical roles in both Int binding and recombination, but it is unclear to what extent these contribute to *attP* identity. Specifically, mutations at positions −20, −21, and −23 strongly interfere with CTD binding to a half site substrate, although these are outside of the minimal length of an *attB* substrate (Figure 1B). So although CTD binds well to a B half site substrate [17] it does not recognize these P mutants as though they are B-type sites. Furthermore, we note that Int binds quite well to single mutants such as T-21C but is poor at recombination, so a plausible explanation is that the conformation of the Int promoter bound at the mutant half site has adopted the conformation as if it were bound to a B-type site, effectively converting the mutant *attP* site into *attL*. Nonetheless, the finding that such single mutant sites can synapse with *attB* (Figure 4) – which *attL* is not able to do – argues strongly against that.

**Figure 5:**
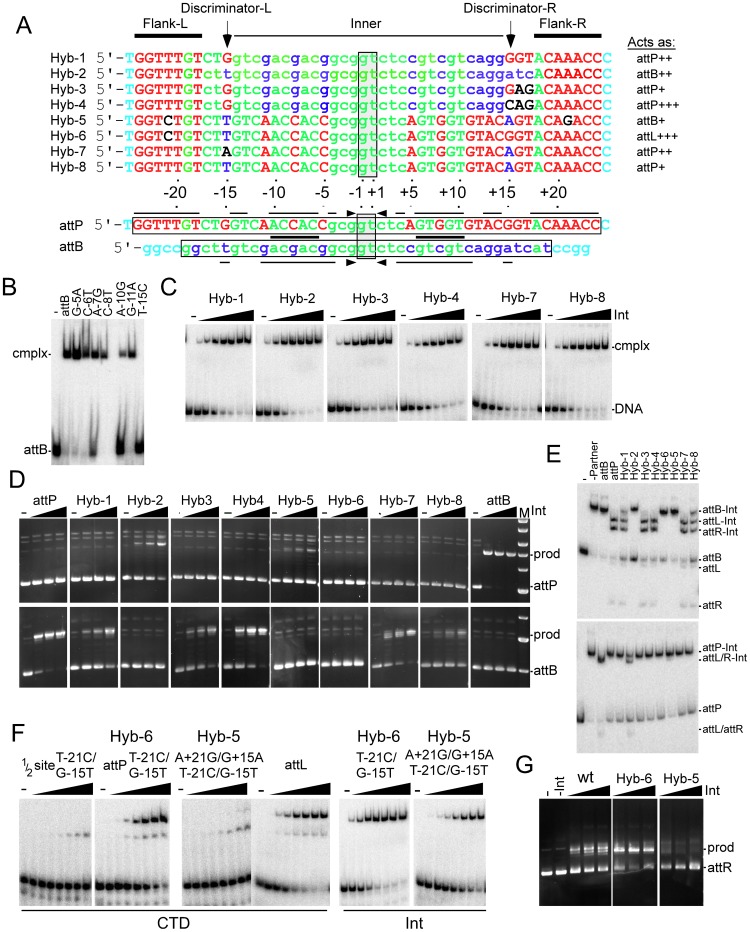
Identifying the determinants of *attP* and *attB* identity. (A) Comparison of *attP* and *attB*, and features of hybrid sites. At the bottom are the top strand sequences of *attP* and *attB* aligned by their central 5′-GT dinucleotides (vertically boxed), with horizontal boxes showing the sequence that are required for recombination. Lines and arrows above and below indicate positions that are conserved in symmetrically related positions in each site, and the thick line between the sequences shows the 5′-ACNAC conserved in all four half sites. The *attP* and *attB* sequences are shown in upper and lower case respectively, and those positions that are common between *attP* and *attB* are shown in green. Positions specific to *attP* or *attB* are shown in red and blue respectively. Bases outside of the minimal size requirements are shown in aqua. Above are shown the sequences of eight hybrid sites with type case and color reflecting derivation from *attB* or *attP*; non-*attB/P* sequences are shown in black. At the top, the site features of the two flanking motifs (Flank-L and Flank-R), the inner motif (Inner) and the ‘Discriminator’ positions at −15 and +15 are shown. The behaviors of the hybrids are shown to the right of each substrates, with ‘+, ‘++’ or ‘+++’ noting its relative strength, with wild-type corresponding to ‘+++’. (B) Separation of complexes formed between CTD and 28 bp half-site DNA containing B half sites with single substitutions as indicated. (C) Separation of complexes formed between Int and different hybrid DNAs as shown. The concentrations of Int are 0, 1.67, 5, 15, 45, 135, 405, 1215 nM (D) Recombination activities are shown for hybrid DNAs using varying concentrations of Int (0, 75, 150, 300 nM). Top and bottom rows show recombination with *attP* and *attB* substrates respectively. Positions of substrate (*attP*/*attB*) and product (prod) are shown. (E) Integration reactions are shown for hybrid sites but using PCR amplified partner DNAs, an asymmetric 213 bp *attB* substrate (top panel) and a symmetrical 106 bp *attP* substrate (bottom panel). The positions of DNAs and complexes are shown. The Int concentration is 300 nM. (F) Complexes formed between CTD and a 50 bp half site substrate of Hybrid-6, full-length Hybrid-5, full length Hybrid-6, and wild-type *attL* are shown. Complexes formed between Int and Hybrids-5 and -6 are also shown. The concentrations of Int and CTD are 0, 1.67, 5, 15, 45, 135, 405, 1215 nM. (G) Hybrid-6 functions as an *attL* substrate. Excision reactions contained both Int and Bxb1 gp47 (1.8 µM); Int concentrations are 35, 70, and 140 nM.

Closer examination of the similarity of the P and B half site sequences show that 13 of the 18 positions are conserved, with differences at positions −5, −8, −11, −15, and −18 (Figure 5A). With the exception of −18, all of these are in symmetrically conserved positions in *attB* (Figure 5A) and are thus candidates for playing roles in determining the identities of *attP* and *attB*, perhaps explaining the failure of CTD to bind to the *attP* flank mutants as though it were a B-type site. To address this, we first determined the impact of single substitutions in these conserved positions of B half site substrates (Figure 5B). The only position with strong inhibition of CTD binding is the position at −15, showing that this is critical for B-type site recognition. We note that the cognate position in *attP* is not symmetrically conserved and is a 5′-GC (top strand-bottom strand) base pair at both −15 and +15 (Figure 5A). Transition mutations in *attP* at these positions have little impact on binding of either CTD or Int, or on recombination (Figure 1, Figure 2, Figure 3).

To define the elements determining site identity, we constructed two hybrid sites (Hybrid-1 and Hybrid-2; Figure 5A). Both contain the inner part of *attB* onto which is added differing lengths of the *attP* flanks; Hybrid-1 and Hybrid-2 have *attP* sequences from −15/+15 and −18/+18 to the ends, respectively (Figure 5A). Int binds remarkably well to both of these hybrid substrates, with affinities of Kd = 13 nM and 7 nM respectively (Figure 5C), similar to binding of Int to *attL* and *attR*, and 4–5 times better than to either *attP* or *attB* [17]. Hybrid-2 retains its ability to recombine as an *attB* substrate – although with somewhat reduced efficiency (Figure 5D, 5E) – but fails to act as an *attP* site. The extreme *attP* flanking sequences thus appear to impair *attB* function, but incompletely. In contrast, Hybrid-1 has completely lost its *attB* identity, but interestingly has gained *attP* identity, recombining with *attB* albeit inefficiently (Figure 5D, 5E). Hybrid-1 and Hybrid-2 differ by only four bases (−15, +15, +16, +17; Figure 5A) and these must then encompass the critical discriminatory positions. Positions 16 and 17 are not symmetrically-related, but are shared between the B and P half sites (Figure 5A) so we constructed two additional substrates; Hybrid-3 adds G+16A/T+17G to Hybrid-1 symmetrizing them with their counterparts in the P and B sites, and Hybrid-4 also symmetrizes the position at +15 (i.e. G+15C). Both hybrids are good Int binding sites (Figure 5C, Kd = 15 nM and 10 nM respectively) and both function as *attP* substrates with Hybrid-4 having near wild-type levels of activity; neither functions as an *attB* site.

These observations suggest that the −15 and +15 positions are discriminator bases playing critical roles in site identity. We therefore tested whether addition of a G-15T substitution (introducing the B-type base pair) to a half-site *attP* substrate containing a T-21C substitution (to which CTD fails to bind; Figure 1C) would restore CTD binding (Figure 5F). We do observe CTD binding to this substrate, although weakly, and a substrate with the same two mutations in both *attP* half sites (Hybrid-5; T-21C/G-15T/G+15A/A+21G) behaves similarly (Figure 5F). However, if the two P-site mutations (T-21C/G-15T) are in a full *attP* context (i.e. with a wild-type P′ site; Hybrid-6), then CTD binds well with efficient formation of complex 2 (Figure 5F). If these two mutations restore a B-type interaction then Hybrid-6 should act as an *attL*-like substrate. We observe that both CTD and Int (Int Kd = 10 nM) bind to Hybrid-6 with similar patterns to *attL* (Figure 5F), and Hybrid-6 is functionally indistinguishable from *attL* for recombination (Figure 5G); it does not function as either *attP* or *attB*. The full Int protein binds slightly less well to Hybrid-5 (Kd = 120 nM) but Hybrid-5 has acquired the ability to function as an *attB* site, albeit inefficiently, and lost the ability to function as *attP* (Figure 5D, 5E). These experiments illustrate the critical roles in the flanking sequences and the −15/+15 base pairs in site identity.

Finally, we constructed two sites that are derivatives of *attP* with G-15A/G+15A and G-15T/G+15A mutations, but with wild-type *attP* flanking sequences (Hybrid-7 and Hybrid-8 respectively). Int binds well to both substrates (Kd = ∼10 nM for both), but neither function as *attB*, and both work only poorly as *attP*, with Hybrid-8 working substantially worse than Hybrid-7 (Figure 5D, 5E). These behaviors are consistent with the interpretation that not only is the T∶A/A∶T (at B and B′ half site respectively) base pair required for *attB* identity, but that it also antagonizes *attP* identity. Likewise, the inability of Hybrid-8 to act as an *attB* site suggests that the *attP* flanking sequence also antagonizes *attB* identity.

## Discussion

Phage-encoded serine integrases show a remarkable selectivity for suitable recombination partner DNAs. This selectivity is inherently related to the biological requirement that these site-specific recombination systems have strong directional control, such that integration and excision do not occur under undesirable circumstances. One consequence of this is that the system must strongly discriminate, for example, between *attP* and the attachment junctions *attL* and *attR*, each of which differs from *attP* by one B-type half site. Because synapsis is a requirement for strand cleavage and is protein-mediated, we assume that different conformations of protein-DNA complexes are the ultimate determinants of site selection. The analysis of *attP* mutants described here provides further support for this model, but also reveals that the *attP* sequence plays a role in controlling post-synaptic events.

**Figure 6:**
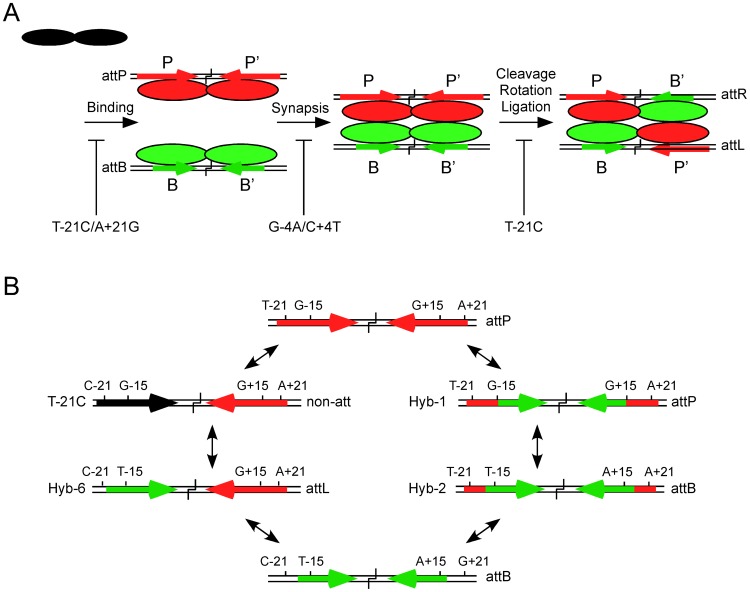
Models for site selection and identity. (A) Bxb1 Int is a dimer in solution (shown in black) and binds to both *attP* and *attB* but is proposed to adopt different conformations when bound to P-type (P, P′; shown in red) or B-type (B, B′; shown in green) half sites. Synapsis occurs only between *attP*-dimer and *attB*-dimer complexes and is required for the subsequent steps of cleavage, rotation, and religation in the product configuration to form *attL* and *attR*. Recombination is highly selective for *attP* and *attB* sites and mutations in *attP* can inhibit different steps in the reaction. Examples include the double mutant T-21C/A+21G that is strongly defective in Int binding, the double mutant G-4A/C+4T that is strongly defective in synapsis, and the T-21C mutant that is defective in a post synaptic step, probably in rotation or ligation. (B) Two possible pathways for inter conversion of *attP* and *attB*. The wild-type *attP* site contains a GC base pair at the discriminator −15 and +15 positions and extreme flanking sequences including a TA base pair in the symmetrically conserved positions −21 and +21. In the right hand pathway, substitution of the inner part of *attP* (from −14 to +14) with that of *attB* (generating Hybrid-1) does not alter its identity as *attP* but reduces its effectiveness as a recombination substrate. However, inclusion of three additional *attB* bases including the critical discriminator TA base pair at positions −15 and +15 (Hybrid-2) switches identity to *attB* although with reduced recombinational activity. Complete removal of the flanks is required for full *attB* function. In the left hand pathway, a single substitution that interferes with binding to the left hand flank of *attP* such as substituting a CG base pair at position −21 (T-21C substrate) results in loss of function as an *attP* site. However, introduction of a TA base pair at the −15 position (Hybrid-6) converts the left half site to B-type identity and the site functions as an *attL* site. Conversion of this substrate to *attB* requires changing the P′ half site to B′ identity with loss of the right *attP* flank and the TA base pair at +15.

Previous analysis showed that the ability to form synaptic complexes is a critical stage in site-selection, although this was based on testing sites to which Int binds but which have substantial sequence differences. The more subtle changes of point mutations show that a block to synapsis can still be observed, such as with the −4/+4 mutant, but that most of the other mutants tested are competent to synapse, even though they may be defective for recombination (Figure 6A). Although we would have predicted that such mutants would be blocked in cleavage, this does not appear to be the case, and at least for single mutations in the P site, cleavage can still occur. The *attP* sequence thus plays an important role in controlling activity, from Int binding through to post-cleavage events (Figure 6A). This mirrors the role of the *attB* sequence in φC31 integration, where mutations interfere with Int binding or synapsis, but also block DNA cleavage [25]. In general, the requirement for satisfying multiple different reactions stages is akin to going through multiple security checks at an airport, needing to pass each one of them before being permitted to board the plane.

The architectures of the Bxb1 *attP* and *attB* sites reflect three types of components (Figure 6B). The first, is the inner part, which we define as encompassing the 28 bp from −14 to +14, and is present in both *attP* and *attB*. Although the sequences of inner-B and inner-P sites differ at a total of nine positions, few appear to play major roles in discrimination between *attP* and *attB*, although most make small contributions to binding. For examples, Hybrid-1, which contains inner-B but with *attP* flanks attached works quite well as an *attP* substrate. Within this region, the −4 and +4 positions are curious as they contribute to CTD binding in spite of being relatively close to the crossover site to which the N-terminal domain must interact, and the −4/+4 double mutant is strongly defective in synapsis, even at concentrations at which Int binds well. We note that double substitutions at positions equivalent to Bxb1 −4/+4 [corresponding to −3/+3 in φC31 [25]] have little impact on binding or recombination in φC31, although changes equivalent to Bxb1 −3/+3 (−2/+2 in φC31) are defective in cleavage [25]. It seems likely that different serine-integrases ‘read’ their sequences in different ways, while sharing in common the process of conformational proof reading at multiple steps in the reaction.

The second architecture feature is the key discriminator positions at −15 and +15 (which we refer to as Discriminator-L and Discriminator-R). The T∶A/A∶T (in B and B′ half sites respectively) base pair is critical for Int binding to *attB*, and for identity as an *attB* site, and when the G-15T mutation is introduced into a half site containing the T-21C, CTD binding is partially restored, presumably with a B-type conformation. This is confirmed by the observation that in the context of the full *attP* site with a wild-type P′ site, Hybrid-6 works with full activity as an *attL* site. Thus, although Int discriminates strongly between *attP* and *attL*, only two base substitutions are needed to interconvert their identities (Figure 6B). Furthermore, repetition of the same two substitutions in the P′ now produces a site with *attB* identity (Hybrid-5) albeit with reduced activity, and eliminates *attP* identity. We note that although inclusion of the T∶A/A∶T base pair (in B and B′ half sites respectively) at both −15 and +15 in *attP* site with proper flanks (Hybrid-8) is not sufficient to switch from *attP* to *attB* identity, it severely impairs *attP* function, and thus antagonizes *attP* identity. Most other substitutions at the −15/+15 positions in *attP* that we tested have little impact on binding or recombination.

The third architectural feature is the two flanking sequences of *attP* that have no counterpart in *attB*. Flank-L and Flank-R (−18 to −24, and +18 to +24, respectively, Figure 5A) are symmetrically conserved and are required for both efficient binding of Int and recombination. Simply adding these to a site with inner-B and *attB* discriminators at −15 and +15 (Hybrid-2) does not prevent the site from acting as *attB*, but considerably impairs it, showing that these not only are required for *attP* function, but are also somewhat anti-*attB*. We note that the flanking sequences of φC31 *attB* are also important for efficient recombination by φC31 Int, although these are all encompassed within the site length requirements for *attP* [25].

In all large serine-recombinase systems in which the site requirements have been examined, *attP* is longer than *attB* [17], [21]–[24], [28], [29], and we therefore propose that the use of the extreme *attP* flanking sequences to confer *attP* identity is a common feature. The use of the −15/+15 discriminator position in other systems is unclear, although we predict that it may be a common site feature, with different systems using different positions for this function. The way in which Int recognizes these features are unclear and no structural information is available. However, we propose that a common DNA binding feature within CTD recognizes the inner parts of both *attP* and *attB*, and we predict that this lies within the N-terminal part [CTDa; [17]] of CTD (Int residues 155–287). Although CTDa alone does not bind DNA efficiently, when connected to the N-terminal catalytic domain (i.e. to include Int residues 1–287) it binds DNA, albeit weakly [17], but recognizes *attP* and *attB* similarly. A zinc-finger motif common to serine integrases – and proposed to be involved in DNA recognition [28] – is located in Bxb1 CTDb at residues 297–354 [17], and we postulate that this specifically recognizes the *attP* flanking sequences.

A striking conclusion from these studies is the simplicity with which site identities can be changed with only a few mutations (Figure 6B). There are likely to be multiple pathways for inter-conversion, and two are shown in Figure 6B. In one pathway, introduction of the single T-21C substitution generates a substrate that binds Int but fails to undergo recombination, and likely fails to act as any type of attachment site. Adding one more substitution (G-15T) converts this into a fully functional *attL* site (Hybrid-6), and introducing the same mutations to convert the P′ site into a B′-like site generates *attB* identity. A second pathway involves addition of the short *attP* flanking sequences to *attB* (Hybrid-2) which then retains *attB* identity but functions poorly. Adding GC base pairs at the −15 and +15 positions then results in a switch to *attP* function. It is noteworthy that none of the inter-conversion pathways we have described generate substrates that can act as both *attP* and *attB*, although this is perhaps not unexpected considering that the key identifiers (*attP* flanks and the discriminators) antagonize one identity while promoting the other. We also recognize that there are clearly additional contributions to site identity and function, as substrates such as Hybrid-7 and Hybrid-8 function as *attP*, but relatively inefficiently. It seems likely that a combination of activities and integration of several site components will be common to other serine-integrase systems, although because there is so much sequence diversity among the sites, often without substantial symmetry and with few positions shared between *attP* and *attB*, understanding site selection and identity in other serine integrase systems will likely require empirical determination.

Serine-integrases are attractive systems for genome manipulation in heterologous systems as well as for construction of synthetic genetic circuits [8], [12], [13], [30]. The Bxb1 system has good attributes for these applications and shows strong site specificity even in large genomic contexts including human, *Drosophila*, and *Plasmodium* genomes [9], [11], [31]. This selectivity derives from multiple proofreading steps in site selection, together with the requirement of key sequences conferring site identity, and understanding these will contribute to the use of serine-integrases for engineering purposes.

## Materials and Methods

### DNA substrates and oligonucleotides

Plasmids pMY1, pMOS-attB, pMOS-attP and pMOS-attR containing 343 bp and 50 bp of *attB*, 200 bp of *attP* and 376 bp of *attR*, respectively, have been described previously [14], [15], [26]. DNA fragments (50 bp) containing wild-type and mutant *attP* sites were prepared by annealing complementary oligonucleotides. Mutant *attP* DNAs containing a single gpInt binding site were prepared by either mutating a half-site (*attP*-mut P half-site or *attP*- mut P′ half-site) or by eliminating a half-site (*attP*-P half-site or *attP*-P′ half-site). These sites are obtained by annealing the necessary pairs of oligonucleotides (Table S2). Mutations were all transitions unless otherwise stated. Suicide substrate *attB* (50 bp) was prepared as described earlier [17] and has a gap 4 nucleotides 5′ of the scissile bond of the top strand (at P site). It is presumed to trap synaptic complexes in which all Int-DNA covalent linkages are formed, but in which religation fails due to loss of the 4-base DNA strand between the gap and the cleavage site on the top strand.

### Bxb1 integrase and CTD overexpression and purification

Bxb1 integrase, CTD and gp47 were purified as described earlier [14], [17]. Stocks of gpInt, CTD and gp47 proteins were diluted as appropriate in 10 mM Tris (pH-7.5), 1 mg/ml Bovine serum albumin (BSA) and 1 mM Dithiothreitol (DTT).

### DNA–binding assays

DNA substrates were prepared by 5′ end labeling of one oligonucleotide of each pair and annealing. Approximately 0.1 pmol of labeled DNA was incubated with either gpInt and CTD in a buffer containing 20 mM Tris (pH-7.5), 25 mM NaCl, 10 mM EDTA, 10 mM Spermidine, 1 mM DTT, and 1 µg Calf Thymus DNA, in a total volume of 10 µl. Reactions were incubated at 37°C for one hour and the protein-DNA complexes separated on a native 5% (unless otherwise stated) polyacrylamide gel at 4°C. Gels were dried, exposed to a phosphorimager screen overnight and scanned (Fuji Phosphoimager). Kd was determined as the Int or CTD concentration in which one half of maximal binding was observed. If multiple complexes were observed the apparent Kd was deduced from the protein concentration at which half of the DNA remained unbound.

### *In vitro* recombination assays

*In vitro* integrative recombination assays were performed as described previously [15] in a recombination buffer containing 20 mM Tris (pH-7.5), 25 mM NaCl, 10 mM EDTA, 10 mM Spermidine and 1 mM DTT in final volume of 10 µl. Reactions using supercoiled pattB DNA contained 0.03 pmol of pMOS and 50 bp of *attP* DNA. The integration reactions were incubated at 37°C for up to 1 h and heat inactivated at 75°C for 15 min. The products were separated by electrophoresis in 0.8% agarose in 1× TBE running buffer and visualized by ethidium bromide staining.

In vitro excision were carried out between 376 bp of *attR* in pMOS-attR and linear *attL* (50 bp) in the above recombination buffer, gpInt and gp47 were added as indicated. The reaction were carried out at 25°C for 2 hours and separated on a 0.8% agarose gel.

For synaptic complex formation and cleavage assays, 5′-end labeled suicide *attB* (50 bp) DNA was incubated with Int and *attP* DNA under the same conditions as for DNA-binding. After 1 hour incubation at 37°C reactions were heat inactivated at 75°C for min 15 min. For cleavage assays reactions were treated with 1 mg/ml Proteinase K and 0.2% SDS at 55°C for 15 min.

## Supporting Information

Figure S1 Binding titrations of Bxb1 CTD and Int to P half site and *attP* mutant substrates. (A) CTD binding to half site substrates containing mutations in the P site as indicated. The concentrations of CTD used are 0, 5, 15, 45, 135, 405 and 1215 nM. (B) Complexes formed between CTD and full length *attP* substrates containing mutations in the P site as indicated are shown. The concentrations of CTD are 0, 15, 45, 135, 405 and 1215 nM. (C) Separation of complexes formed with Int and *attP* DNA containing mutations in P site are shown. The concentrations of Int used are 0, 15, 45, 135, 405 and 1215 nM.
(PDF)

Figure S2 Binding titration of Int and CTD to *attP* substrates with P′ site mutations. (A) Separation of complexes formed by CTD with *attP* substrates containing mutations in P′ site. (B) Separation of complexes formed by Int with *attP* substrates containing mutations in P′ site are shown. The concentrations of Int and CTD used are 0, 15, 45, 135, 405 and 1215 nM.
(PDF)

Figure S3 Recombination of mutant *attP* substrates. (A–C) Recombination activities of *attP* substrates having mutations either in P (A), P′ (B), or in both half sites (C) (double mutants). The Int concentrations in panel (A) are 0, 18.75, 37.5, 75, 150 nM and in panel (B–C) are 0, 37.5, 75, 150, 300 nM.
(PDF)

Figure S4 Synaptic complex formation by mutant *attP* substrates. *attP* mutant substrates are incubated with radiolabeled suicide *attB* substrate and varying concentration of gpInt to form synaptic complexes. The concentrations of gpInt are 0, 45, 135, 405, 1215 nM.
(PDF)

Table S1 Binding affinities by Bxb1 Int for P′ mutant *attP* sites.
(PDF)

Table S2 Oligonucleotides used in this study.
(PDF)
