# Supplementary figures and images for: Attachment Site Selection and Identity in Bxb1 Serine Integrase-Mediated Site-Specific Recombination
Source: PLoS Genet. 2013 May 2;9(5):e1003490. doi: 10.1371/journal.pgen.1003490 (PMC3642061; doi:10.1371/journal.pgen.1003490)

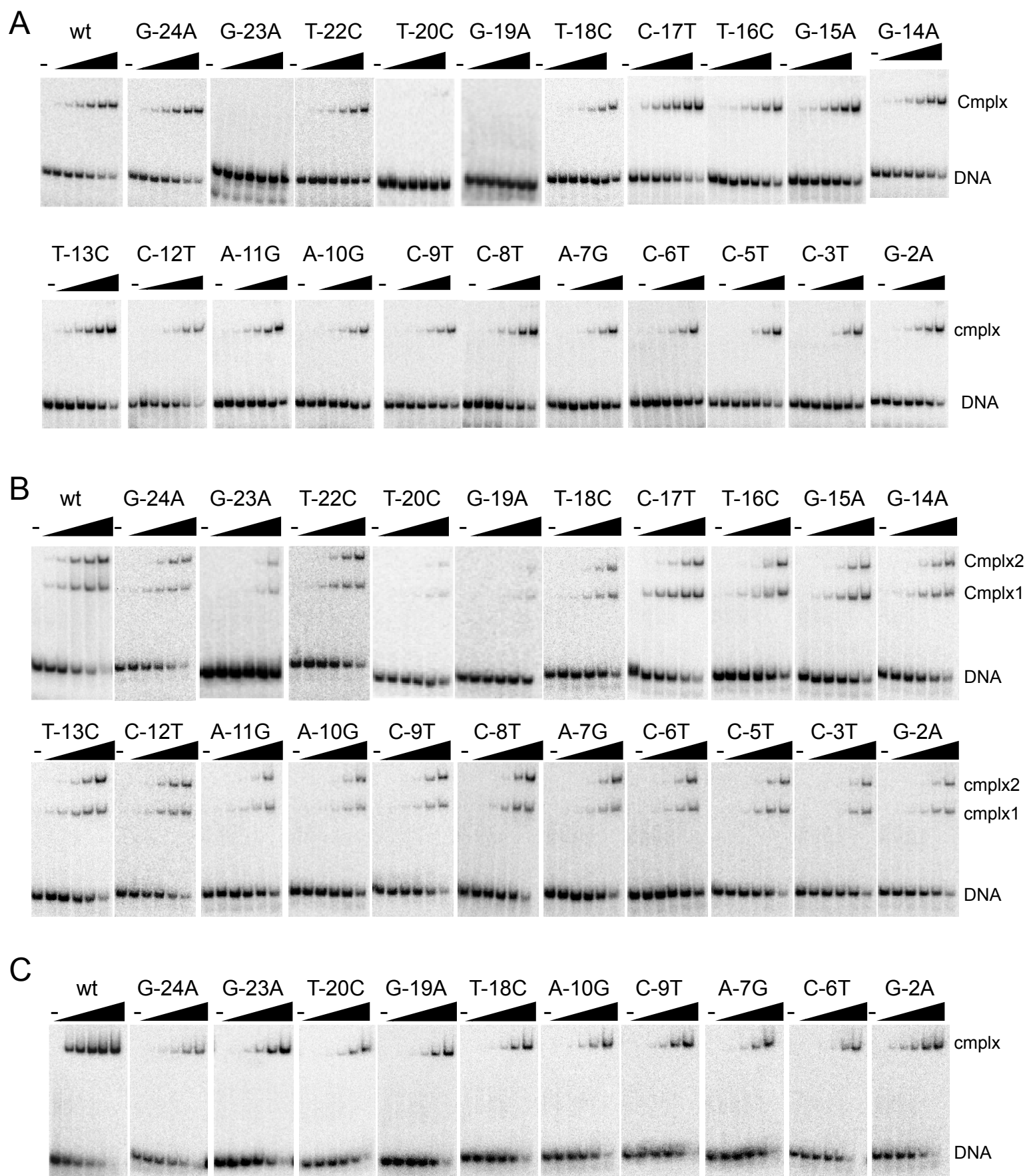

Figure S1

Supplement: Figure S1 — Binding titrations of Bxb1 CTD and Int to P half site and attP mutant substrates. (A) CTD binding to half site substrates containing mutations in the P site as indicated. The concentrations of CTD used are 0, 5, 15, 45, 135, 405 and 1215 nM. (B) Complexes formed between CTD and full length attP substrates containing mutations in the P site as indicated are shown. The concentrations of CTD are 0, 15, 45, 135, 405 and 1215 nM. (C) Separation of complexes formed with Int and attP DNA containing mutations in P site are shown. The concentrations of Int used are 0, 15, 45, 135, 405 and 1215 nM. (PDF) [file pgen.1003490.s001.pdf]

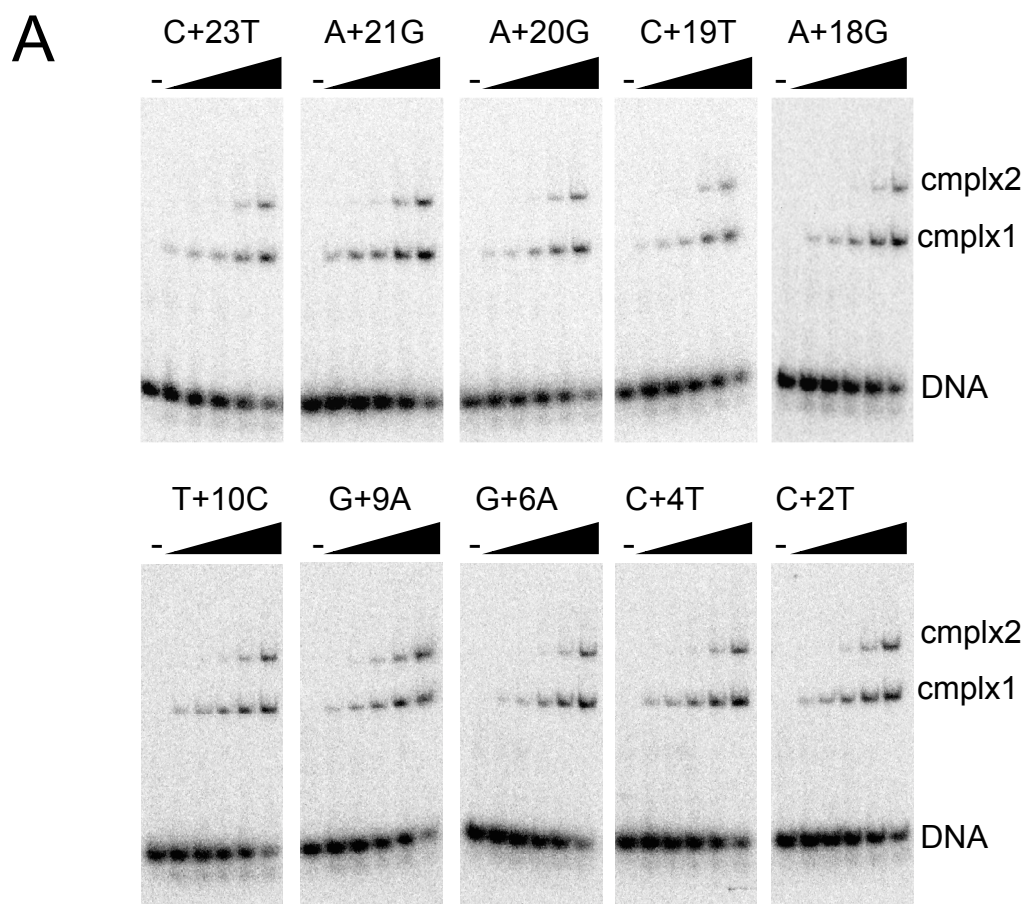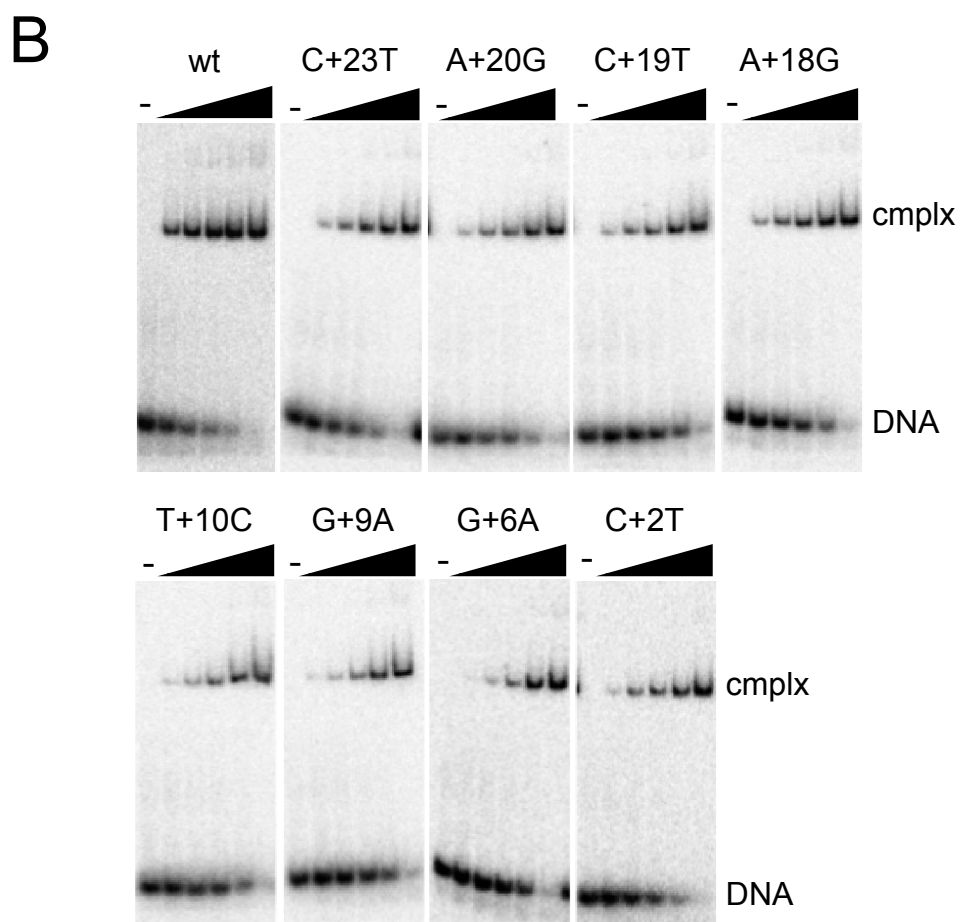

Figure S2

Supplement: Figure S2 — Binding titration of Int and CTD to attP substrates with P′ site mutations. (A) Separation of complexes formed by CTD with attP substrates containing mutations in P′ site. (B) Separation of complexes formed by Int with attP substrates containing mutations in P′ site are shown. The concentrations of Int and CTD used are 0, 15, 45, 135, 405 and 1215 nM. (PDF) [file pgen.1003490.s002.pdf]

A

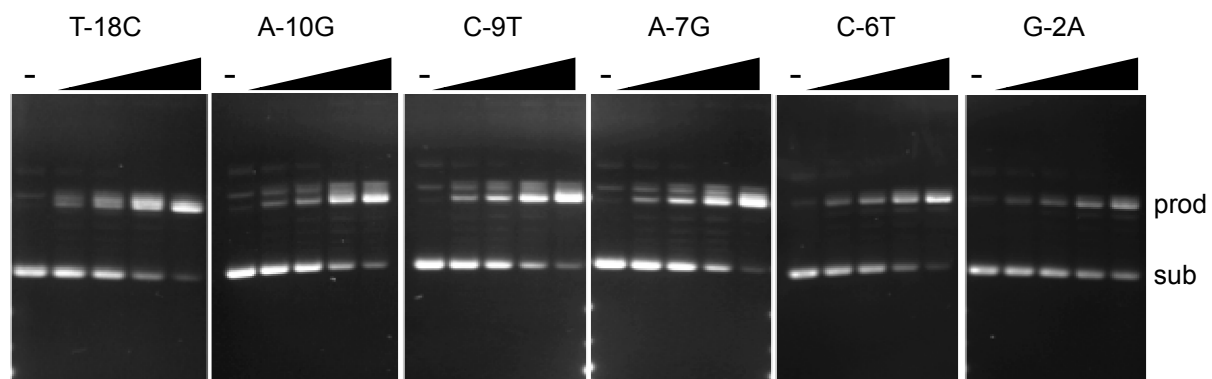

B

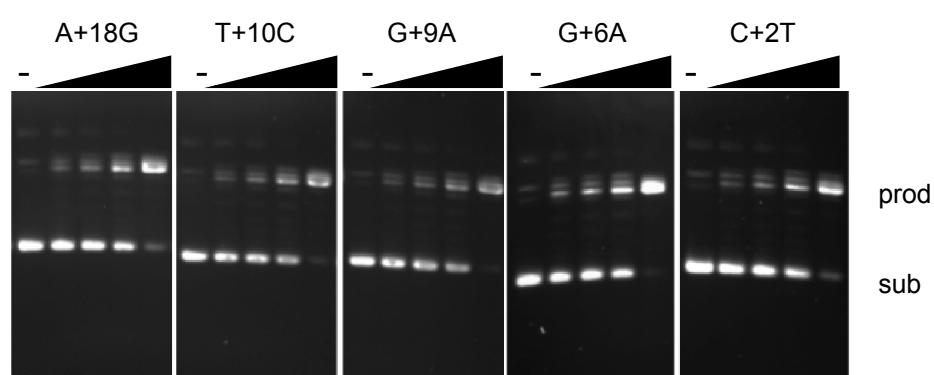

C

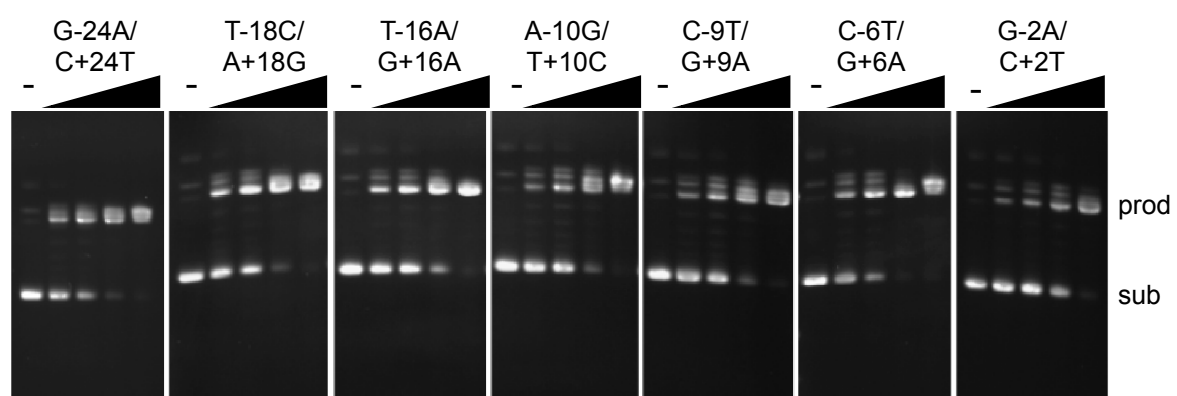

Figure S3

Supplement: Figure S3 — Recombination of mutant attP substrates. (A–C) Recombination activities of attP substrates having mutations either in P (A), P′ (B), or in both half sites (C) (double mutants). The Int concentrations in panel (A) are 0, 18.75, 37.5, 75, 150 nM and in panel (B–C) are 0, 37.5, 75, 150, 300 nM. (PDF) [file pgen.1003490.s003.pdf]

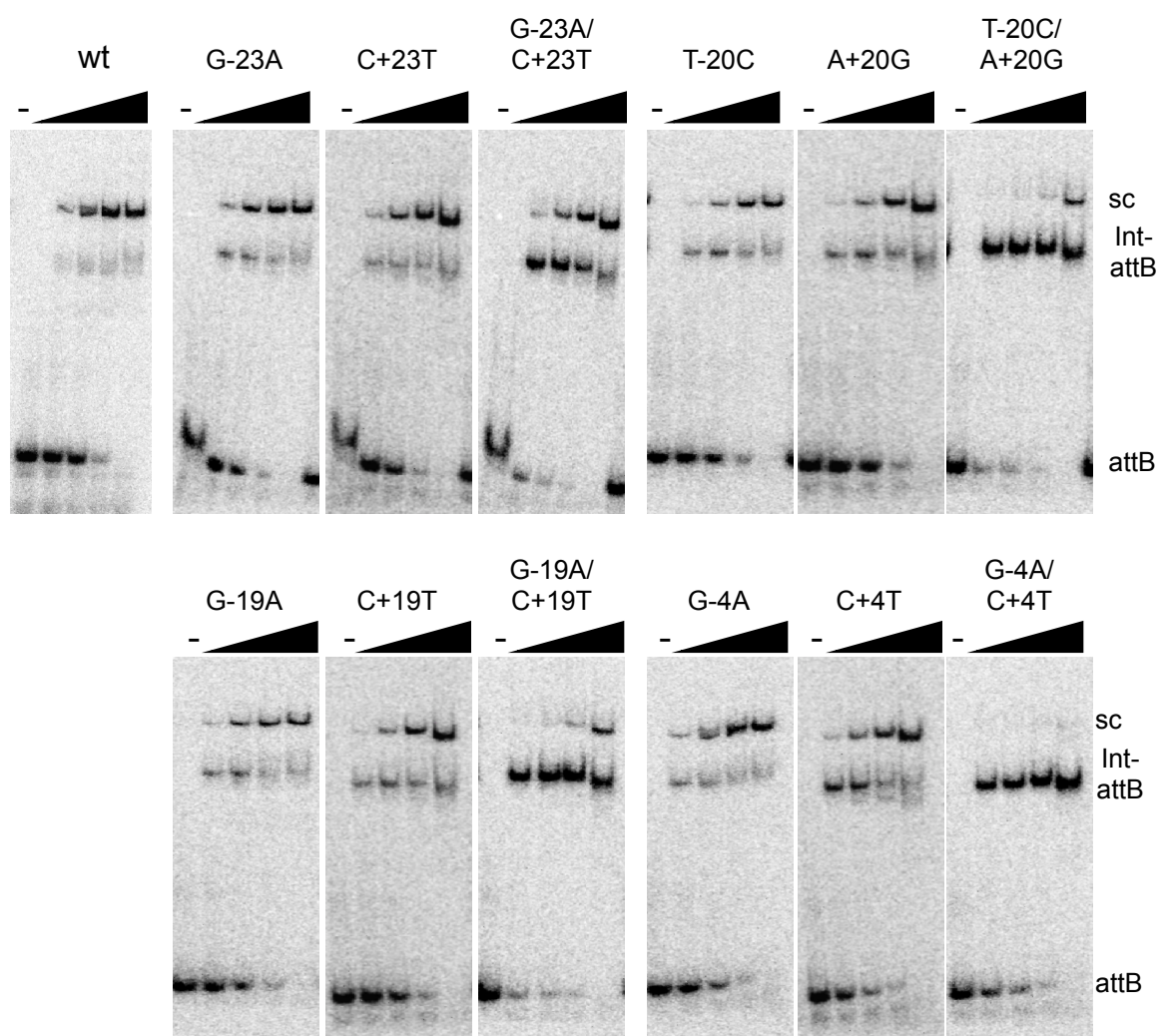

Figure S4

Supplement: Figure S4 — Synaptic complex formation by mutant attP substrates. attP mutant substrates are incubated with radiolabeled suicide attB substrate and varying concentration of gpInt to form synaptic complexes. The concentrations of gpInt are 0, 45, 135, 405, 1215 nM. (PDF) [file pgen.1003490.s004.pdf]
