# Supplementary material for: Attachment Site Selection and Identity in Bxb1 Serine Integrase-Mediated Site-Specific Recombination
Source: PLoS Genet. 2013 May 2;9(5):e1003490. doi: 10.1371/journal.pgen.1003490 (PMC3642061; doi:10.1371/journal.pgen.1003490)
Supplement: Table S1 — Binding affinities by Bxb1 Int for P′ mutant attP sites. (PDF) [file pgen.1003490.s005.pdf]

Table S1. Binding affinities by Bxb1 Int for P’ mutant *attP* sites

**Mutation**[1]	**Kd of Int binding to full site (nM)**[2]
Wild type	60
C+2T	150
C+4T	290
G+6A	250
G+9A	320
T+10C	280
A+18G	200
C+19T	300
A+20G	200
A+21G	150
C+23T	175

[1]Mutations are denoted as the wild-type base, the position, and the altered base
[2]50 bp full site substrates have a mutant P’ half site and a wild-type P half site
