# Supplementary material for: Attachment Site Selection and Identity in Bxb1 Serine Integrase-Mediated Site-Specific Recombination
Source: PLoS Genet. 2013 May 2;9(5):e1003490. doi: 10.1371/journal.pgen.1003490 (PMC3642061; doi:10.1371/journal.pgen.1003490)
Supplement: Table S2 — Oligonucleotides used in this study. (PDF) [file pgen.1003490.s006.pdf]

Table S2: Oligonucleotides used in this study.

attP	**Mutations in P-site of *attP***
wt	5′ TGGTTTGTCTGGTCAACCACCGCGGTCTCAGTGGTGTACGGTACAAACCC 3′
	5′ GGGTTTGTACCGTACACCACTGAGACCGCGGTGGTTGACCAGACAAACCA 3′
-2	5′ TGGTTTGTCTGGTCAACCACCGCAGTCTCAGTGGTGTACGGTACAAACCC 3′
	5′ GGGTTTGTACCGTACACCACTGAGACTGCGGTGGTTGACCAGACAAACCA 3′
-3	5′ TGGTTTGTCTGGTCAACCACCGTGGTCTCAGTGGTGTACGGTACAAACCC 3′
	5′ GGGTTTGTACCGTACACCACTGAGACCACGGTGGTTGACCAGACAAACCA 3′
-4	5′ TGGTTTGTCTGGTCAACCACCACGGTCTCAGTGGTGTACGGTACAAACCC 3′
	5′ GGGTTTGTACCGTACACCACTGAGACCGTGGTGGTTGACCAGACAAACCA 3′
-5	5′ TGGTTTGTCTGGTCAACCACTGCGGTCTCAGTGGTGTACGGTACAAACCC 3′
	5′ GGGTTTGTACCGTACACCACTGAGACCGCAGTGGTTGACCAGACAAACCA 3′
-6	5′ TGGTTTGTCTGGTCAACCATCGCGGTCTCAGTGGTGTACGGTACAAACCC 3′
	5′ GGGTTTGTACCGTACACCACTGAGACCGCGATGGTTGACCAGACAAACCA 3′
-7	5′ TGGTTTGTCTGGTCAACCGCCGCGGTCTCAGTGGTGTACGGTACAAACCC 3′
	5′ GGGTTTGTACCGTACACCACTGAGACCGCGGCGGTTGACCAGACAAACCA 3′
-8	5′ TGGTTTGTCTGGTCAACTACCGCGGTCTCAGTGGTGTACGGTACAAACCC 3′
	5′ GGGTTTGTACCGTACACCACTGAGACCGCGGTAGTTGACCAGACAAACCA 3′
-9	5′ TGGTTTGTCTGGTCAATCACCGCGGTCTCAGTGGTGTACGGTACAAACCC 3′
	5′ GGGTTTGTACCGTACACCACTGAGACCGCGGTGATTGACCAGACAAACCA 3′
-10	5′ TGGTTTGTCTGGTCAGCCACCGCGGTCTCAGTGGTGTACGGTACAAACCC 3′
	5′ GGGTTTGTACCGTACACCACTGAGACCGCGGTGGCTGACCAGACAAACCA 3′
-11	5′ TGGTTTGTCTGGTCGACCACCGCGGTCTCAGTGGTGTACGGTACAAACCC 3′
	5′ GGGTTTGTACCGTACACCACTGAGACCGCGGTGGTCGACCAGACAAACCA 3′
-12	5′ TGGTTTGTCTGGTTAACCACCGCGGTCTCAGTGGTGTACGGTACAAACCC 3′
	5′ GGGTTTGTACCGTACACCACTGAGACCGCGGTGGTTAACCAGACAAACCA 3′
-13	5′ TGGTTTGTCTGGCCAACCACCGCGGTCTCAGTGGTGTACGGTACAAACCC 3′
	5′ GGGTTTGTACCGTACACCACTGAGACCGCGGTGGTTGGCCAGACAAACCA 3′
-14	5′ TGGTTTGTCTGATCAACCACCGCGGTCTCAGTGGTGTACGGTACAAACCC 3′
	5′ GGGTTTGTACCGTACACCACTGAGACCGCGGTGGTTGATCAGACAAACCA 3′
-15	5′ TGGTTTGTCTAGTCAACCACCGCGGTCTCAGTGGTGTACGGTACAAACCC 3′
	5′ GGGTTTGTACCGTACACCACTGAGACCGCGGTGGTTGACTAGACAAACCA 3′
-16	5′ TGGTTTGTCCGGTCAACCACCGCGGTCTCAGTGGTGTACGGTACAAACCC 3′
	5, GGGTTTGTACCGTACACCACTGAGACCGCGGTGGTTGACCGGACAAACCA 3′
-17	5′ TGGTTTGTTTGGTCAACCACCGCGGTCTCAGTGGTGTACGGTACAAACCC 3′
	5, GGGTTTGTACCGTACACCACTGAGACCGCGGTGGTTGACCAAACAAACCA 3′
-18	5′ TGGTTTGCCTGGTCAACCACCGCGGTCTCAGTGGTGTACGGTACAAACCC 3′
	5′ GGGTTTGTACCGTACACCACTGAGACCGCGGTGGTTGACCAGGCAAACCA 3′
-19	5′ TGGTTTATCTGGTCAACCACCGCGGTCTCAGTGGTGTACGGTACAAACCC 3′
	5′ GGGTTTGTACCGTACACCACTGAGACCGCGGTGGTTGACCAGATAAACCA 3′
-20	5′ TGGTTCGTCTGGTCAACCACCGCGGTCTCAGTGGTGTACGGTACAAACCC 3′
	5′ GGGTTTGTACCGTACACCACTGAGACCGCGGTGGTTGACCAGACGAACCA 3′
-21	5′ TGGTCTGTCTGGTCAACCACCGCGGTCTCAGTGGTGTACGGTACAAACCC 3′
	5′ GGGTTTGTACCGTACACCACTGAGACCGCGGTGGTTGACCAGACAGACCA 3′
-22	5′ TGGCTTGTCTGGTCAACCACCGCGGTCTCAGTGGTGTACGGTACAAACCC 3′
	5′ GGGTTTGTACCGTACACCACTGAGACCGCGGTGGTTGACCAGACAAGCCA 3′
-23	5′ TGATTTGTCTGGTCAACCACCGCGGTCTCAGTGGTGTACGGTACAAACCC 3′
	5′ GGGTTTGTACCGTACACCACTGAGACCGCGGTGGTTGACCAGACAAATCA 3′
-24	5′ TAGTTTGTCTGGTCAACCACCGCGGTCTCAGTGGTGTACGGTACAAACCC 3′
	5′ GGGTTTGTACCGTACACCACTGAGACCGCGGTGGTTGACCAGACAAACTA 3′

***attP*-mut P** **_attP_-mut P half site**

**half site**	
wt(LH)	5′ CCGTGATGACCTGTGTCTTCGTGGTTTGTCTGGTCAACCACCGCGGTCTC 3′
	5′ GAGACCGCGGTGGTTGACCAGACAAACCACGAAGACACAGGTCATCACGG 3′
-2	5′ CCGTGATGACCTGTGTCTTCGTGGTTTGTCTGGTCAACCACCGCAGTCTC 3′
	5′ GAGACTGCGGTGGTTGACCAGACAAACCACGAAGACACAGGTCATCACGG 3′
-3	5′ CCGTGATGACCTGTGTCTTCGTGGTTTGTCTGGTCAACCACCGTGGTCTC 3′
	5′ GAGACCACGGTGGTTGACCAGACAAACCACGAAGACACAGGTCATCACGG 3′
-4	5′ CCGTGATGACCTGTGTCTTCGTGGTTTGTCTGGTCAACCACCACGGTCTC 3′
	5′ GAGACCGTGGTGGTTGACCAGACAAACCACGAAGACACAGGTCATCACGG 3′
-5	5′ CCGTGATGACCTGTGTCTTCGTGGTTTGTCTGGTCAACCACTGCGGTCTC 3′
	5′ GAGACCGCAGTGGTTGACCAGACAAACCACGAAGACACAGGTCATCACGG 3′
-6	5′ CCGTGATGACCTGTGTCTTCGTGGTTTGTCTGGTCAACCATCGCGGTCTC 3′
	5′ GAGACCGCGATGGTTGACCAGACAAACCACGAAGACACAGGTCATCACGG 3′
-7	5′ CCGTGATGACCTGTGTCTTCGTGGTTTGTCTGGTCAACCGCCGCGGTCTC 3′
	5′ GAGACCGCGGCGGTTGACCAGACAAACCACGAAGACACAGGTCATCACGG 3′
-8	5′ CCGTGATGACCTGTGTCTTCGTGGTTTGTCTGGTCAACTACCGCGGTCTC 3′
	5′ GAGACCGCGGTAGTTGACCAGACAAACCACGAAGACACAGGTCATCACGG 3′
-9	5′ CCGTGATGACCTGTGTCTTCGTGGTTTGTCTGGTCAATCACCGCGGTCTC 3′
	5′ GAGACCGCGGTGATTGACCAGACAAACCACGAAGACACAGGTCATCACGG 3′
-10	5′ CCGTGATGACCTGTGTCTTCGTGGTTTGTCTGGTCAGCCACCGCGGTCTC 3′
	5′ GAGACCGCGGTGGCTGACCAGACAAACCACGAAGACACAGGTCATCACGG 3′
-11	5′ CCGTGATGACCTGTGTCTTCGTGGTTTGTCTGGTCGACCACCGCGGTCTC 3′
	5′ GAGACCGCGGTGGTCGACCAGACAAACCACGAAGACACAGGTCATCACGG 3′
-12	5′ CCGTGATGACCTGTGTCTTCGTGGTTTGTCTGGTTAACCACCGCGGTCTC 3′
	5′ GAGACCGCGGTGGTTAACCAGACAAACCACGAAGACACAGGTCATCACGG 3′
-13	5′ CCGTGATGACCTGTGTCTTCGTGGTTTGTCTGGCCAACCACCGCGGTCTC 3′
	5′ GAGACCGCGGTGGTTGGCCAGACAAACCACGAAGACACAGGTCATCACGG 3′
-14	5′ CCGTGATGACCTGTGTCTTCGTGGTTTGTCTGATCAACCACCGCGGTCTC 3′
	5′ GAGACCGCGGTGGTTGATCAGACAAACCACGAAGACACAGGTCATCACGG 3′
-15	5′ CCGTGATGACCTGTGTCTTCGTGGTTTGTCTAGTCAACCACCGCGGTCTC 3′
	5′ GAGACCGCGGTGGTTGACTAGACAAACCACGAAGACACAGGTCATCACGG 3′
-16	5′ CCGTGATGACCTGTGTCTTCGTGGTTTGTCCGGTCAACCACCGCGGTCTC 3′
	5′ GAGACCGCGGTGGTTGACCGGACAAACCACGAAGACACAGGTCATCACGG 3′
-17	5′ CCGTGATGACCTGTGTCTTCGTGGTTTGTTTGGTCAACCACCGCGGTCTC 3′
	5′ GAGACCGCGGTGGTTGACCAAACAAACCACGAAGACACAGGTCATCACGG 3′
-18	5′ CCGTGATGACCTGTGTCTTCGTGGTTTGCCTGGTCAACCACCGCGGTCTC 3′
	5′ GAGACCGCGGTGGTTGACCAGGCAAACCACGAAGACACAGGTCATCACGG 3′
-19	5′ CCGTGATGACCTGTGTCTTCGTGGTTTATCTGGTCAACCACCGCGGTCTC 3′
	5′ GAGACCGCGGTGGTTGACCAGATAAACCACGAAGACACAGGTCATCACGG 3′
-20	5′ CCGTGATGACCTGTGTCTTCGTGGTTCGTCTGGTCAACCACCGCGGTCTC 3′
	5′ GAGACCGCGGTGGTTGACCAGACGAACCACGAAGACACAGGTCATCACGG 3′
-21	5′ CCGTGATGACCTGTGTCTTCGTGGTCTGTCTGGTCAACCACCGCGGTCTC 3′
	5′ GAGACCGCGGTGGTTGACCAGACAGACCACGAAGACACAGGTCATCACGG 3′
-22	5′ CCGTGATGACCTGTGTCTTCGTGGCTTGTCTGGTCAACCACCGCGGTCTC 3′
	5′ GAGACCGCGGTGGTTGACCAGACAAGCCACGAAGACACAGGTCATCACGG 3′
-23	5′ CCGTGATGACCTGTGTCTTCGTGATTTGTCTGGTCAACCACCGCGGTCTC 3′
	5′ GAGACCGCGGTGGTTGACCAGACAAATCACGAAGACACAGGTCATCACGG 3′
-24	5′ CCGTGATGACCTGTGTCTTCGTAGTTTGTCTGGTCAACCACCGCGGTCTC 3′
	5′ GAGACCGCGGTGGTTGACCAGACAAACTACGAAGACACAGGTCATCACGG 3′
***attP*-mut P′ half site**	***attP*-mut P′ half site**
wt	5′ gcggtctcAGTGGTGTACGGTACAAACCCATGAGAGCCCTGGTAGTCATC 3′
	5′ GATGACTACCAGGGCTCTCATGGGTTTGTACCGTACACCACTgagaccgc 3′
+2	5′ gcggtTtcAGTGGTGTACGGTACAAACCCATGAGAGCCCTGGTAGTCATC 3′
	5′ GATGACTACCAGGGCTCTCATGGGTTTGTACCGTACACCACTgaAaccgc 3′
+3	5′ gcggtcCcAGTGGTGTACGGTACAAACCCATGAGAGCCCTGGTAGTCATC 3′
	5′ GATGACTACCAGGGCTCTCATGGGTTTGTACCGTACACCACTgGgaccgc 3′

+4	5′ gcggtctTAGTGGTGTACGGTACAAACCCATGAGAGCCCTGGTAGTCATC 3′
	5′ GATGACTACCAGGGCTCTCATGGGTTTGTACCGTACACCACTAagaccgc 3′
+5	5′ gcggtctcGGTGGTGTACGGTACAAACCCATGAGAGCCCTGGTAGTCATC 3′
	5′ GATGACTACCAGGGCTCTCATGGGTTTGTACCGTACACCACCgagaccgc 3′
+6	5′ gcggtctcAaTGGTGTACGGTACAAACCCATGAGAGCCCTGGTAGTCATC 3′
	5′ GATGACTACCAGGGCTCTCATGGGTTTGTACCGTACACCAtTgagaccgc 3′
+7	5′ gcggtctcAGCGGTGTACGGTACAAACCCATGAGAGCCCTGGTAGTCATC 3′
	5′ GATGACTACCAGGGCTCTCATGGGTTTGTACCGTACACCGCTgagaccgc 3′
+8	5′ gcggtctcAGTaGTGTACGGTACAAACCCATGAGAGCCCTGGTAGTCATC 3′
	5′ GATGACTACCAGGGCTCTCATGGGTTTGTACCGTACACtACTgagaccgc 3′
+9	5′ gcggtctcAGTGaTGTACGGTACAAACCCATGAGAGCCCTGGTAGTCATC 3′
	5′ GATGACTACCAGGGCTCTCATGGGTTTGTACCGTACAtCACTgagaccgc 3′
+10	5′ gcggtctcAGTGGcGTACGGTACAAACCCATGAGAGCCCTGGTAGTCATC 3′
	5′ GATGACTACCAGGGCTCTCATGGGTTTGTACCGTACgCCACTgagaccgc 3′
+11	5′ gcggtctcAGTGGTaTACGGTACAAACCCATGAGAGCCCTGGTAGTCATC 3′
	5′ GATGACTACCAGGGCTCTCATGGGTTTGTACCGTAtACCACTgagaccgc 3′
+12	5′ gcggtctcAGTGGTGcACGGTACAAACCCATGAGAGCCCTGGTAGTCATC 3′
	5′ GATGACTACCAGGGCTCTCATGGGTTTGTACCGTgCACCACTgagaccgc 3′
+13	5′ gcggtctcAGTGGTGTgCGGTACAAACCCATGAGAGCCCTGGTAGTCATC 3′
	5′ GATGACTACCAGGGCTCTCATGGGTTTGTACCGcACACCACTgagaccgc 3′
+14	5′ gcggtctcAGTGGTGTAtGGTACAAACCCATGAGAGCCCTGGTAGTCATC 3′
	5′ GATGACTACCAGGGCTCTCATGGGTTTGTACCaTACACCACTgagaccgc 3′
+15	5′ gcggtctcAGTGGTGTACaGTACAAACCCATGAGAGCCCTGGTAGTCATC 3′
	5′ GATGACTACCAGGGCTCTCATGGGTTTGTACtGTACACCACTgagaccgc 3′
+16	5′ gcggtctcAGTGGTGTACGaTACAAACCCATGAGAGCCCTGGTAGTCATC 3′
	5′ GATGACTACCAGGGCTCTCATGGGTTTGTAtCGTACACCACTgagaccgc 3′
+17	5′ gcggtctcAGTGGTGTACGGcACAAACCCATGAGAGCCCTGGTAGTCATC 3′
	5′ GATGACTACCAGGGCTCTCATGGGTTTGTgCCGTACACCACTgagaccgc 3′
+18	5′ gcggtctcAGTGGTGTACGGTgCAAACCCATGAGAGCCCTGGTAGTCATC 3′
	5′ GATGACTACCAGGGCTCTCATGGGTTTGcACCGTACACCACTgagaccgc 3′
+19	5′ gcggtctcAGTGGTGTACGGTAtAAACCCATGAGAGCCCTGGTAGTCATC 3′
	5′ GATGACTACCAGGGCTCTCATGGGTTTaTACCGTACACCACTgagaccgc 3′
+20	5′ gcggtctcAGTGGTGTACGGTACgAACCCATGAGAGCCCTGGTAGTCATC 3′
	5′ GATGACTACCAGGGCTCTCATGGGTTcGTACCGTACACCACTgagaccgc 3′
+21	5′ gcggtctcAGTGGTGTACGGTACAgACCCATGAGAGCCCTGGTAGTCATC 3′
	5′ GATGACTACCAGGGCTCTCATGGGTcTGTACCGTACACCACTgagaccgc 3′
+22	5′ gcggtctcAGTGGTGTACGGTACAAgCCCATGAGAGCCCTGGTAGTCATC 3′
	5′ GATGACTACCAGGGCTCTCATGGGcTTGTACCGTACACCACTgagaccgc 3′
+23	5′ gcggtctcAGTGGTGTACGGTACAAAtCCATGAGAGCCCTGGTAGTCATC 3′
	5′ GATGACTACCAGGGCTCTCATGGaTTTGTACCGTACACCACTgagaccgc 3′
+24	5′ gcggtctcAGTGGTGTACGGTACAAACtCATGAGAGCCCTGGTAGTCATC 3′
	5′ GATGACTACCAGGGCTCTCATGaGTTTGTACCGTACACCACTgagaccgc 3′

attP	**Mutation in P′-site of *attP***
+2	5′ TGGTTTGTCTGGTCAACCACCGCGGTTTCAGTGGTGTACGGTACAAACCC 3′
	5′ GGGTTTGTACCGTACACCACTGAAACCGCGGTGGTTGACCAGACAAACCA 3′
+4	5′ TGGTTTGTCTGGTCAACCACCGCGGTCTTAGTGGTGTACGGTACAAACCC 3′
	5′ GGGTTTGTACCGTACACCACTAAGACCGCGGTGGTTGACCAGACAAACCA 3′
+6	5′ TGGTTTGTCTGGTCAACCACCGCGGTCTCAATGGTGTACGGTACAAACCC 3′
	5′ GGGTTTGTACCGTACACCATTGAGACCGCGGTGGTTGACCAGACAAACCA 3′
+9	5′ TGGTTTGTCTGGTCAACCACCGCGGTCTCAGTGATGTACGGTACAAACCC 3′
	5′ GGGTTTGTACCGTACATCACTGAGACCGCGGTGGTTGACCAGACAAACCA 3′
+10	5′ TGGTTTGTCTGGTCAACCACCGCGGTCTCAGTGGCGTACGGTACAAACCC 3′
	5′ GGGTTTGTACCGTACGCCACTGAGACCGCGGTGGTTGACCAGACAAACCA 3′
+18	5′ TGGTTTGTCTGGTCAACCACCGCGGTCTCAGTGGTGTACGGTGCAAACCC 3′
	5′ GGGTTTGCACCGTACACCACTGAGACCGCGGTGGTTGACCAGACAAACCA 3′

+19	5′ TGGTTTGTCTGGTCAACCACCGCGGTCTCAGTGGTGTACGGTATAAACCC 3′
	5′ GGGTTTATACCGTACACCACTGAGACCGCGGTGGTTGACCAGACAAACCA 3′
+20	5′ TGGTTTGTCTGGTCAACCACCGCGGTCTCAGTGGTGTACGGTACGAACCC 3′
	5′ GGGTTCGTACCGTACACCACTGAGACCGCGGTGGTTGACCAGACAAACCA 3′
+21	5′ TGGTTTGTCTGGTCAACCACCGCGGTCTCAGTGGTGTACGGTACAGACCC 3′
	5′ GGGTCTGTACCGTACACCACTGAGACCGCGGTGGTTGACCAGACAAACCA 3′
+23	5′ TGGTTTGTCTGGTCAACCACCGCGGTCTCAGTGGTGTACGGTACAAATCC 3′
	5′ GGATTTGTACCGTACACCACTGAGACCGCGGTGGTTGACCAGACAAACCA 3′
**Double mutation**	**Mutation in P and P′-site of *attP***
-/+2	5′ TGGTTTGTCTGGTCAACCACCGCAGTTTCAGTGGTGTACGGTACAAACCC 3′
	5′ GGGTTTGTACCGTACACCACTGAAACTGCGGTGGTTGACCAGACAAACCA 3′
-/+4	5′ TGGTTTGTCTGGTCAACCACCACGGTCTTAGTGGTGTACGGTACAAACCC 3′
	5′ GGGTTTGTACCGTACACCACTAAGACCGTGGTGGTTGACCAGACAAACCA 3′
-/+6	5′ TGGTTTGTCTGGTCAACCATCGCGGTCTCAATGGTGTACGGTACAAACCC 3′
	5′ GGGTTTGTACCGTACACCATTGAGACCGCGATGGTTGACCAGACAAACCA 3′
-/+9	5′ TGGTTTGTCTGGTCAATCACCGCGGTCTCAGTGATGTACGGTACAAACCC 3′
	5′ GGGTTTGTACCGTACATCACTGAGACCGCGGTGATTGACCAGACAAACCA 3′
-/+10	5′ TGGTTTGTCTGGTCAGCCACCGCGGTCTCAGTGGCGTACGGTACAAACCC 3′
	5′ GGGTTTGTACCGTACGCCACTGAGACCGCGGTGGCTGACCAGACAAACCA 3′
-/+18	5′ TGGTTTGCCTGGTCAACCACCGCGGTCTCAGTGGTGTACGGTGCAAACCC 3′
	5′ GGGTTTGCACCGTACACCACTGAGACCGCGGTGGTTGACCAGGCAAACCA 3′
-/+19	5′ TGGTTTATCTGGTCAACCACCGCGGTCTCAGTGGTGTACGGTATAAACCC 3′
	5′ GGGTTTATACCGTACACCACTGAGACCGCGGTGGTTGACCAGATAAACCA 3′
-/+20	5′ TGGTTCGTCTGGTCAACCACCGCGGTCTCAGTGGTGTACGGTACGAACCC 3′
	5′ GGGTTCGTACCGTACACCACTGAGACCGCGGTGGTTGACCAGACGAACCA 3′
-/+21	5′ TGGTCTGTCTGGTCAACCACCGCGGTCTCAGTGGTGTACGGTACAGACCC 3′
	5′ GGGTCTGTACCGTACACCACTGAGACCGCGGTGGTTGACCAGACAGACCA 3′
-/+23	5′ TGATTTGTCTGGTCAACCACCGCGGTCTCAGTGGTGTACGGTACAAATCC 3′
	5′ GGATTTGTACCGTACACCACTGAGACCGCGGTGGTTGACCAGACAAATCA 3′
attB	***attB*- suicide substrate**
	5′ TCGGCCGGCTTGTCGACGACGG 3′
	5′ CGGTCTCCGTCGTCAGGATCATCCGGGC 3′
	5′ GCCCGGATGATCCTGACGACGGAGACCGCCGTCGTCGACAAGCCGGCCGA 3′
